# Niche and range dynamics of Tasmanian blue gum (*Eucalyptus globulus* Labill.), a globally cultivated invasive tree

**DOI:** 10.1002/ece3.9305

**Published:** 2022-09-17

**Authors:** Runyao Cao, Xiang Gong, Jianmeng Feng, Rujing Yang

**Affiliations:** ^1^ Department of Life Science and Agronomy Dali University Dali China

**Keywords:** cultivated invasive tree, ecological niche model, niche conservatism, niche dynamics, range expansion, Tasmanian blue gum

## Abstract

The ecological niche concept has provided insights into various areas in ecology and biogeography. Although there remains much controversy regarding whether species niches are conserved across space and time, many recent studies have suggested that invasive species conserve their climatic niche between native and introduced ranges; however, whether the climatic niche of cultivated invasive species, whose niches are strongly affected by human activities, are conserved between native and introduced ranges remains unclear. Additionally, the range dynamics of invasive species in their native and introduced regions have not been extensively studied. Here, we investigated the niche and range dynamics of Tasmanian blue gum (*Eucalyptus globulus* Labill.), a globally cultivated invasive tree, using ecological niche models and niche dynamic analyses. The most important factors affecting the niche changes between native and introduced Tasmanian blue gum were max temperature of the warmest month and precipitation of the wettest month. The climate niche was not conserved between introduced and native range Tasmanian blue gum; moreover, the niche area of the former was ca. 7.4 times larger than that of the latter, as introduced Tasmanian blue gum could survive in hotter, colder, wetter, and drier climates. In addition, the potential range of introduced Tasmanian blue gum was ca. 32 times larger than that of its native counterpart. Human introduction and cultivation may play a key role in the niche and range expansion of introduced Tasmanian blue gum. Given that small increases in niche area can result in large range expansions, the niche expansion of an invasive species could be used to evaluate invasion risk, which might even be more sensitive than range expansions.

## Graphical abstract

The climate niche was conserved between introduced and native Tasmanian blue gum, though the niche breadth of the former was ca. 1.96 times wider than that of the latter. Small increases in niche breadth can result in large range expansions of Tasmanian blue gum.The niche expansion of an invasive species could be used to evaluate invasion risk, which might even be more sensitive than range expansion.

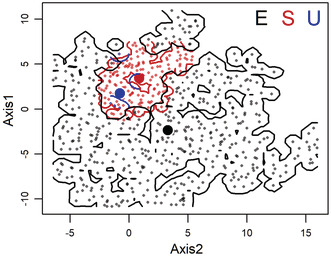

## 1 INTRODUCTION

The ecological niche concept, which describes the range of conditions under which a particular species can exist (Hutchinson, 1957), is one of the pillars of ecological theory. It links species distributions to environmental variables, has been a major focus of contemporary ecology and biogeography (Guisan et al., 2014; Sax et al., 2013; Sexton et al., 2017), and provides insight into various topics, such as biodiversity patterns (Rolland et al., 2018; Wiens & Graham, 2005), patterns of species coexistence (Kraft et al., 2015; Tedersoo et al., 2020), speciation (Sexton et al., 2017; Warren et al., 2008), and range shifts of species under the background of global change (Gong et al., 2020; Liu, Wang, et al., 2020). Global biomes have experienced unprecedented changes due to anthropogenic activities (e.g., habitat loss, introduction of invasive species) and climate change since the start of the Anthropocene (Newbold et al., 2015; Sax et al., 2013). Ecological niche models (ENMs) can be used to predict the potential distribution of species, including shifts under global change scenarios (Booth, 2016; Booth et al., 2014; Liu, Wang, et al., 2020; Peterson et al., 2011), and this information can aid the development of biodiversity conservation management strategies (Peterson & Holt, 2003; Sax et al., 2013). One of the key underlying assumptions of ENMs is niche conservatism (i.e., species niches remain stable or change slowly across space and time), which is thought to cause species to occur in areas with similar environmental conditions in new geographical areas (e.g., following introduction to a new region) or in different periods.

There has been much recent controversy over whether species niches are conserved across space and time (i.e., niche conservatism hypothesis; Guisan et al., 2014; Peterson, 2011; Sexton et al., 2017; Valladares et al., 2014), as evidence from various studies has provided support for (e.g., Lauzeral et al., 2011; Parravicini et al., 2015) and against (e.g., Beukema et al., 2018; Strubbe et al., 2013) this hypothesis. This lack of consensus reduces the robustness of ENMs for predicting species distributions across space and time (Liu, Wang, et al., 2020) and restricts our ability to predict species responses to global changes (Wiens et al., 2009).

Biological invasions or species introductions provide an excellent opportunity to explore this issue. Although several studies of the niche dynamics of invasive species have been conducted in recent years, much controversy remains. For example, in a study of 815 introduced plant species, Atwater et al. (2018) documented considerable climatic niche shifts in more than 65% of species. By contrast, Petitpierre et al. (2012) and Liu, Wolter, et al. (2020) found that niche shifts were rare in plant invaders. The niche conservatism of invasive species thus requires further study.

Guo et al. (2013) and Guisan et al. (2014) argued that conclusions regarding niche conservatism can vary depending on the approaches used. Three types of approaches have been used to explore niche dynamics: ENM, univariate, and ordination approaches. Although none of these approaches are suitable across all contexts, ordination approach, such as the COUE scheme (Centroid shift, Overlap, Unfilling, and Expansion; Broennimann et al., 2012; Petitpierre et al., 2012), has become the most robust approach for exploring niche dynamics (Atwater et al., 2018; Datta et al., 2019; Guisan et al., 2014; Jourdan et al., 2021). Liu, Wolter, et al. (2020) used ordination approach to investigate the niche dynamics of 434 alien invasive species and found that niche spaces were conserved in ca. 60% of the invasive species analyzed. Most previous studies have focused on the niche shifts of common invasive species or introduced species (e.g., Jourdan et al., 2021; Liu, Wang, et al., 2020); by contrast, few studies of the niche dynamics of invasive species under human cultivation have been conducted. Thus, whether the climatic niches of cultivated invasive species, whose niches are strongly modified by human activities, are conserved remains unclear.

Studies of niche dynamics have generally suggested that the shifts in the niches of species caused by environmental changes might be closely associated with species range shifts (Aguilee et al., 2016; Davies et al., 2019; Li et al., 2014). For example, the contraction of niches might be accompanied by reductions in range size and vice versa (Bernard et al., 2021; Jourdan et al., 2021; Kafaei et al., 2021; Kambach et al., 2019; Rather et al., 2021; Slatyer et al., 2013). Range shifts of invasive species have received increased research attention in recent years. Liu, Wang, et al. (2020) showed that the fall armyworm (*Spodoptera frugiperda* J.E. Smith), a globally invasive species, might undergo a considerable range expansion under the background of future climate change using maximum entropy model (MaxEnt) ENMs. Using biomod2, an ecological modeling platform, including generalized boosting model (GBM), generalized additive model (GAM), classification tree analysis (CTA), artificial neural network (ANN), flexible discriminant analysis (FDA), and random forest (RF), Gong et al. (2020), found that climate change might have double‐edged effects on the range sizes of invasive species, resulting in range contraction and expansion of two invasive species. Most of these previous studies have assumed that niche space is stable when predicting species distributions under global change scenarios, and this can have a substantial effect on the predictions of ENMs (Jourdan et al., 2021), and therefore, studies of range shifts under scenarios of niche shifts are critically important.

Tasmanian blue gum (*Eucalyptus globulus* Labill.), which is native to southern Victoria (Australia), Tasmania, and the Bass Strait Islands (Jordan et al., 1999), has become one of the most widely planted exotic trees since it was first introduced to other regions from Australia in the mid‐20th century (Potts et al., 2004; Rejmánek & Richardson, 2011), and plantations of this species expanded rapidly worldwide because of its excellent wood quality and fiber characteristics (Potts et al., 2004). Potts et al. (2004) estimated that there were over 2.3 million hectares of Tasmanian blue gum cultivated in the tropical, subtropical, and Mediterranean regions of all continents. Tasmanian blue gum has become an important part of the hardwood forestry industry in several countries (e.g., China, Ethiopia, Uruguay, Chile, and Australia; Potts et al., 2004). Despite the worldwide distribution of its plantations and its economic importance, Tasmanian blue gum is still considered invasive in various parts of the world (Rejmánek & Richardson, 2013), possibly because its secondary metabolites impair ecosystem function (Castro‐Diez et al., 2010), and it can increase the risk of forest fires (Fernandes et al., 2011; Silva et al., 2011). In the USA, this species poses the highest invasion risk among 38 invasive *Eucalyptus* species, partly because of its ability to rapidly proliferate following its introduction and plantation (Gordon et al., 2012). Seedling recruitment is several times higher in Portugal where Tasmanian blue gum plantations are widespread than in its native range, and this contributes to its high invasion risk to local biomes (Águas et al., 2017; Catry et al., 2015). A weed risk assessment of a set of alien species in Spain and Australia identified Tasmanian blue gum as an invasive species that poses high environmental risk (Gassó et al., 2010; Larcombe et al., 2013; Lazarides et al., 1997). Invasions of Tasmanian blue gum can have significant effects on natural forest ecosystems by modifying canopy structure, critical habitat features for various animal species, and ecosystem processes (Asner et al., 2008; Brooks et al., 2004; Williams & Wardle, 2005). Although study of the niche dynamics and potential range of Tasmanian blue gum could aid the development of approaches to reduce the threat that this species poses to global ecological security, to the best of our knowledge, no studies of the global niche and range dynamics of Tasmanian blue gum have been conducted to date.

In this study, we aimed to explore the niche and range changes between native and introduced Tasmanian blue gum and identify the factors controlling the niche dynamics of Tasmanian blue gum. The results of our study provide new information that will help managers and policymakers develop strategies to mitigate the negative impacts of this globally invasive tree. Our findings also provide general insights into the niche and range changes between native and introduced species.

## 2 MATERIAL AND METHODS

### 2.1 Global occurrence records of Tasmanian blue gum

**FIGURE 1:**
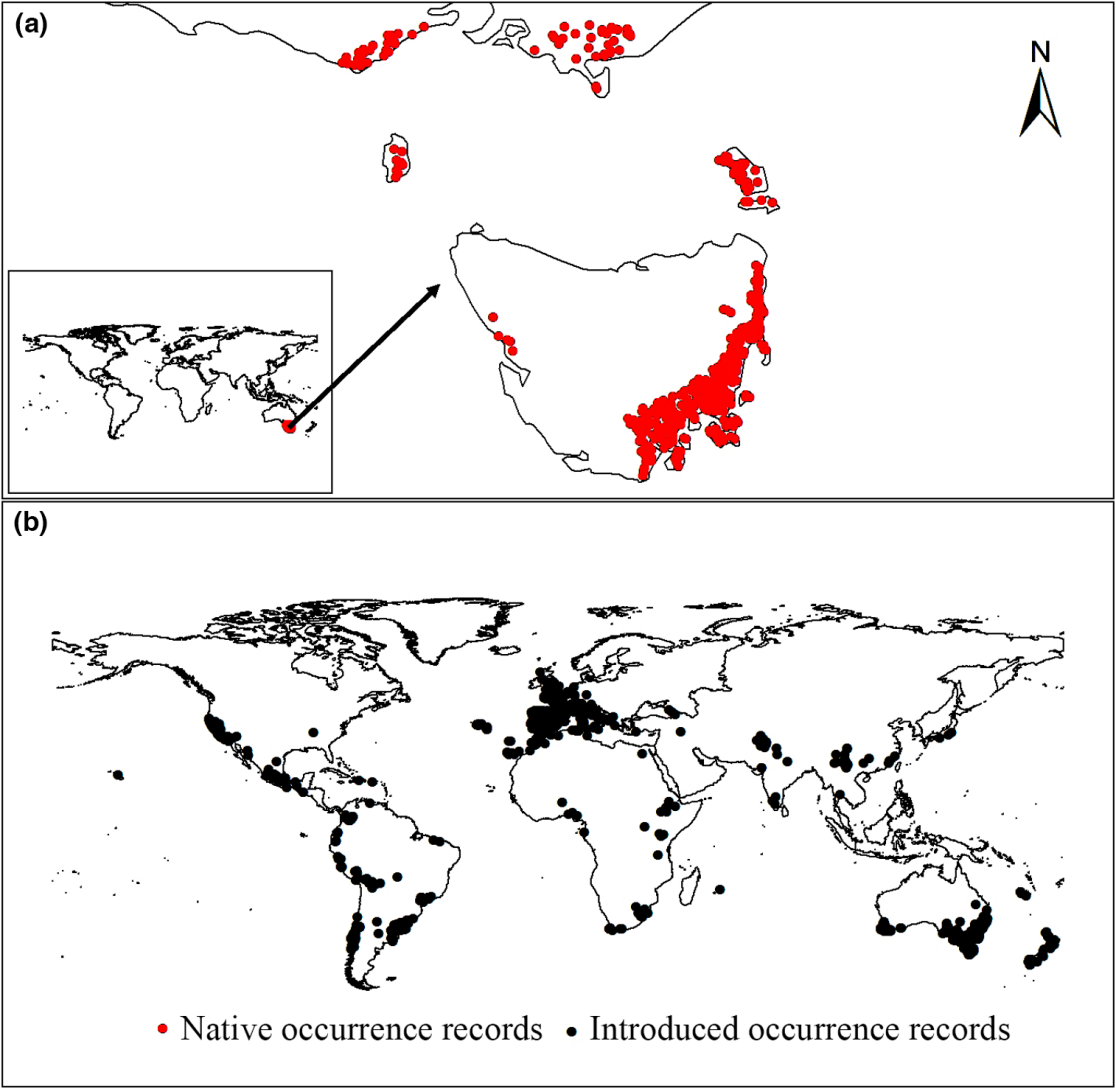
Native and introduced occurrence records of Tasmanian blue gum. (a and b) demonstrated occurrence records of Tasmanian blue gum in native (red) and introduced (black) ranges, respectively. We spatially rarefied occurrence records with a radius of 10 km to reduce spatial autocorrelations, and we obtained 2488 occurrence records, including 398 and 2090 occurrence records from native and introduction ranges, respectively.

We compiled a global dataset of occurrence records of Tasmanian blue gum using the two following sources: (1) the Global Biodiversity Information Facility (GBIF) database (https://www.gbif.org/, accessed on Nov 21, 2020), from which we retrieved 18,303 distinct occurrence records with geographical coordinates under 10 km uncertainty, and (2) an extensive and systematic literature survey that generated 695 distinct records with geographical coordinates (Appendix S1). This is the largest dataset of the occurrence records of Tasmanian blue gum assembled to date. Next, we divided our large dataset into two sub‐datasets based on the distributions of the introduced and native ranges of Tasmanian blue gum delimited by Larcombe et al. (2013): the native range dataset, which consisted of 2222 occurrence records, and the introduced range dataset, which consisted of 16,776 occurrence records. We thus used the SDM toolbox (Brown, 2014; Brown et al., 2017) to spatially rarefy occurrence records with a radius of 10 km to reduce spatial autocorrelation. After spatial rarefaction, we were left with 398 and 2090 occurrence records for the native and introduced range datasets, respectively (Figure 1).

### 2.2 Predictors in the ENMs

As our major goal was to investigate climatic niche shifts, as well as make comparisons with previous studies, we used a dataset of 19 bioclimatic factors that was retrieved from the Worldclim database at a spatial resolution of 5 arc‐minutes (ca. 10 km) for 1970–2000 (Fick & Hijmans, 2017). Strong collinearity among predictors may cause over‐prediction in ENMs. Following a method proposed by Gong et al. (2020) and Liu, Wang, et al. (2020), we accounted for collinearity by retrieving the values of 19 predictor (bioclimatic factor) layers for each occurrence record and then using Pearson's correlation analyses to identify pairs of predictors showing strong collinearity; we retained variables with *r* ≤ 0.70, which is a well‐validated threshold commonly used in niche modeling (Appendix S1; Dormann et al., 2013). Through biomod v.2.0 (Thuiller et al., 2009), an ENM platform, we inputted 19 bioclimatic predictors to generate preliminary ENMs and determined the relative importance values of each predictor (Appendix S2) using seven different algorithms in the biomod v.2.0 platform (Thuiller et al., 2009) to generate ENMs, including MaxEnt, GAM, FDA, GBM, CTA, RF, and ANN. Predictors showing lower importance values or values of zero in each pair of predictors exhibiting strong collinearity were removed from subsequent analyses. This process stopped when no strong collinearity among the predictors was detected (Appendix S3). The retained predictors (Appendix S3) were input into final ENMs to project the potential range of Tasmanian blue gum.

### 2.3 Assessing niche dynamics

Niche spaces of native and introduced Tasmanian blue gum and their dynamics were calculated using a gaussian kernel density estimator with the hypervolume R package with default parameters (see Blonder et al., 2018 for details). Two principal component (PC) axes for 19 bioclimatic factors generated through principal component analysis were used to delimit the two‐dimensional niche spaces of Tasmanian blue gum in its native and introduced range, as well as to identify the most important climatic predictors underlying niche dynamics. A kernel density function was then used to estimate the smoothed density of occurrences in environmental space (Blonder et al., 2018).

The niche conservatism hypothesis was confirmed when the niche space of Tasmanian blue gum in its introduced range was smaller than that in its native range, or when Tasmanian blue gum in its introduced and native range occupies similar positions in niche space (Broennimann et al., 2007; Pearman et al., 2008). In our study, the niche space occupied by native and introduced Tasmanian blue gum were classified as niche expansion (E), niche unfilling (U), and niche stability (S). E indicates the niche space occupied only by Tasmanian blue gum in its introduced range; U indicates the niche space occupied only by Tasmanian blue gum in its native range; and S indicates the proportion of niche space occupied by Tasmanian blue gum in both its introduced and native range. The sum of E and S was the niche space of Tasmanian blue gum in its introduced range. The sum of S and U was the niche space of Tasmanian blue gum in its native range. We used the ratio of the niche space of Tasmanian blue gum in its introduced range to that of Tasmanian blue gum in its native range to measure the changes in niche area:

$$\mathrm{NAR}=\frac{{\mathrm{NA}}_{i}}{{\mathrm{NA}}_{n}}$$

where NAR, NA_*n*_, and NA_*i*_ are the niche area ratio, the niche area of Tasmanian blue gum in its native range, and the niche area of Tasmanian blue gum in its introduced range, respectively.

Sørensen's similarity index (SI) was used to measure changes in niche position:

$$\mathrm{SI}=\frac{2S}{\left({\mathrm{NA}}_{n}+{\mathrm{NA}}_{i}\right)}$$

Following Pearman et al. (2008) and Broennimann et al. (2007), if NAR > 1 and SI < 0.5, then the niche conservatism hypothesis was rejected.

We then used independent‐samples *t*‐tests to compare the mean values of the most important climatic predictors derived from the trimmed occurrence records (i.e., max temperature of the warmest month and precipitation of the wettest month) loaded on PC1 and PC2.

### 2.4 Projecting potential ranges of Tasmanian blue gum

Using the retained predictors (Appendix S3), we applied seven different algorithms in the biomod v.2.0 platform (Thuiller et al., 2009) to generate ENMs, including MaxEnt, GAM, FDA, GBM, CTA, RF, and ANN (Thuiller et al., 2009). To account for the potentially spurious effects of the different ENM methods, we only retained ENMs with true skill statistics (TSS; Allouche et al., 2006) that were greater than 0.7 or areas under the receiver operating characteristic (ROC) curve (AUC; Fielding & Bell, 1997) that were greater than 0.8 (e.g., Gallien et al., 2012). We then used an ensemble approach to obtain the central tendency of the ENMs through a weight proportional to the TSS that was assigned to each model's projection (Araujo & New, 2007). To meet the requirement of presence and pseudo‐absences (PAs) in ENMs, we generated two sets of PAs by retrieving random points at a global scale. As suggested by Barbet‐Massin et al. (2012), we randomly selected 1000 Pas if the number of presence records was less than or equal to 1000, and equal weightings were assigned to presences and Pas. Finally, we used the threshold of the sensitivity‐specificity sum maximization approach (MSS threshold) to determine the potential native and introduced ranges of Tasmanian blue gum.

### 2.5 Evaluation of ENMs

A four‐time repeated split sampling approach was used to evaluate the predictive performance of ENMs; 70% of the occurrence records were used to build the ENMs, and the remaining 30% were used to evaluate the performance of the ENMs. Three different evaluation metrics were used, including area under the ROC curve (AUC; Fielding & Bell, 1997), the Kappa coefficient (Cohen, 1960), and TSS (Allouche et al., 2006). The thresholds for reliable ensemble models were as follows: AUC above 0.8, Kappa above 0.6, and TSS above 0.7, respectively.

### 2.6 Assessing range dynamics

We compared the spatial patterns of habitat suitability (i.e., high suitability (>0.6), moderate suitability (0.4–0.6), low suitability (0.2–0.4), and not suitable (<0.2)) between introduced and native niches. We also explored differences in the area of suitable habitats in native and introduced ranges (suitable habitats), as well as their spatial patterns. We used Mapcurve, a quantitative goodness‐of‐fit (GOF) method proposed by Hargrove et al. (2006) that shows the degree of spatial concordance between two categorical maps, to compare the spatial patterns of the suitable and unsuitable portions of the potential range of native and introduced Tasmanian blue gum, which was estimated as follows:

$$\mathrm{GOF}=\sum_{i=1}^{n}\frac{C_{i}}{B_{i}+C_{i}}*\frac{C_{i}}{A_{i}+C_{i}}$$

where *A* and *B* are the numbers of grid cells in the potential native and introduced ranges, respectively, and *C* and *n* are the intersection of these two ranges and the number of categories in the maps, respectively. A similar analysis was conducted on the non‐potential native and introduced ranges.

## 3 RESULTS

### 3.1 Factors affecting niche changes between native and introduced Tasmanian blue gum

**FIGURE 2:**
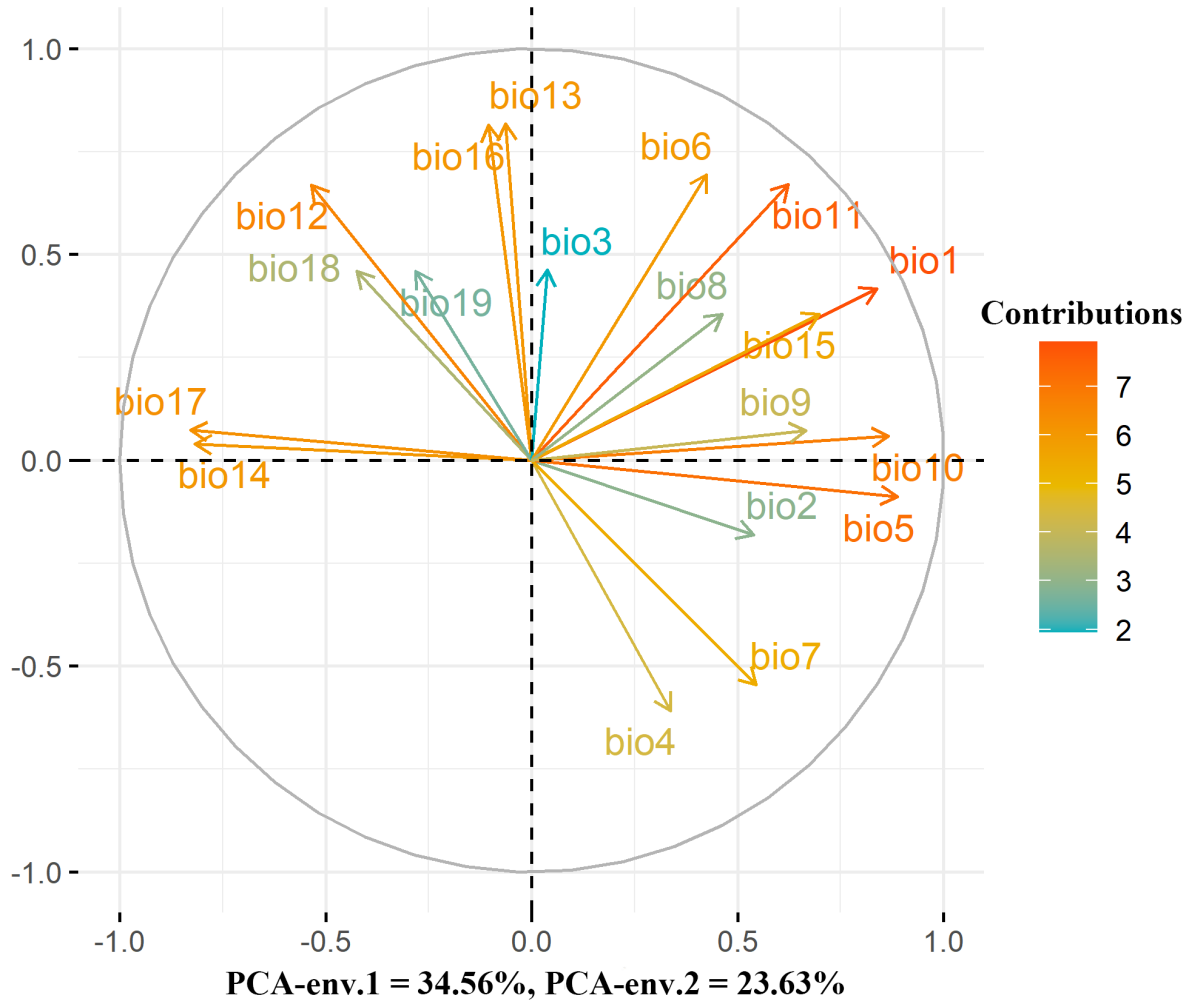
Principal component analysis used to delimit two‐dimensional niche space. Two PC axes accounted for 58.19% of the variability among all climatic variables. The 1st and 2nd axis of the explained 34.56% and 23.63% of the variance, respectively. The 1st axis of the PCA mainly reflected thermal axis, and the most important closely related variable was Bio5 (max temperature of the warmest month; with load being −0.887), followed by Bio10 (mean temperature of the warmest quarter; −0.866), respectively. The 2nd axis can be interpreted as water axis, and the most important responsible variable was Bio13 (precipitation of the wettest month; 0.817), followed by Bio16 (precipitation of the wettest quarter; 0.814), respectively. Bio1: Annual mean temperature; Bio2: Mean diurnal range; bio3: Isothermality; bio4: Temperature seasonality; bio5: Max temperature of warmest month; Bio6: Min temperature of coldest month; Bio7: Temperature annual range; Bio8: Mean temperature of wettest quarter; Bio9: Mean temperature of driest quarter; Bio10: Mean temperature of warmest quarter; Bio11: Mean temperature of coldest quarter; Bio12: Annual precipitation; Bio13: Precipitation of wettest month; Bio14: Precipitation of driest month; Bio15: Precipitation seasonality; Bio16: Precipitation of wettest quarter; Bio17: Precipitation of driest quarter; Bio18: Precipitation of warmest quarter; Bio19: Precipitation of coldest quarter.

A two‐dimensional niche area delimited two PC axes accounting for 58.19% of the variation among all climatic variables (Figure 2); specifically, PC1 and PC2 explained 34.56% and 23.63% of the variance, respectively (Figure 2). PC1 was closely associated with thermal variables, and the variables that were most heavily loaded on PC1 were max temperature of the warmest month (−0.887) and mean temperature of the warmest quarter (−0.866; Appendix S5, Figure 2). PC2 was primarily a water axis, and the variable that was most heavily loaded on PC2 was precipitation of the wettest month (0.817) and precipitation of the wettest quarter (0.814; Appendix S5, Figure 2).

### 3.2 Niche changes between native and introduced Tasmanian blue gum

**FIGURE 3:**
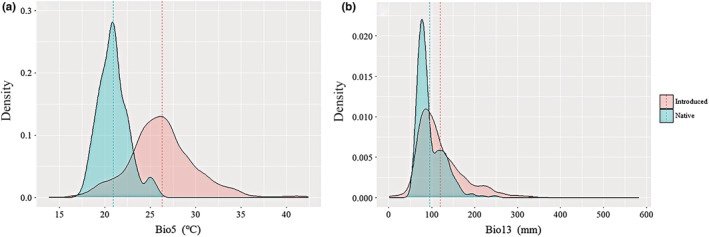
Comparisons of the climatic predictors derived from native and introduced occurrence records. The mean values of the mean temperature of the warmest quarter (Bio5) and precipitation of the wettest month (Bio13) derived from native occurrence records were significantly greater than those from introduced ones (both *p* < .01, a, b).

The main niche shift observed in introduced Tasmanian blue gum relative to its native niche was an increase along PC1 and PC2 of niche area (the thermal and water axes, respectively). Independent‐samples *t*‐tests revealed that max temperature of the warmest month based on the introduced occurrence data was significantly greater than that based on the native occurrence data (*P* = 0.001); the same was also the case for precipitation of the wettest month (*P* = 0.001; Figure 3).

**FIGURE 4:**
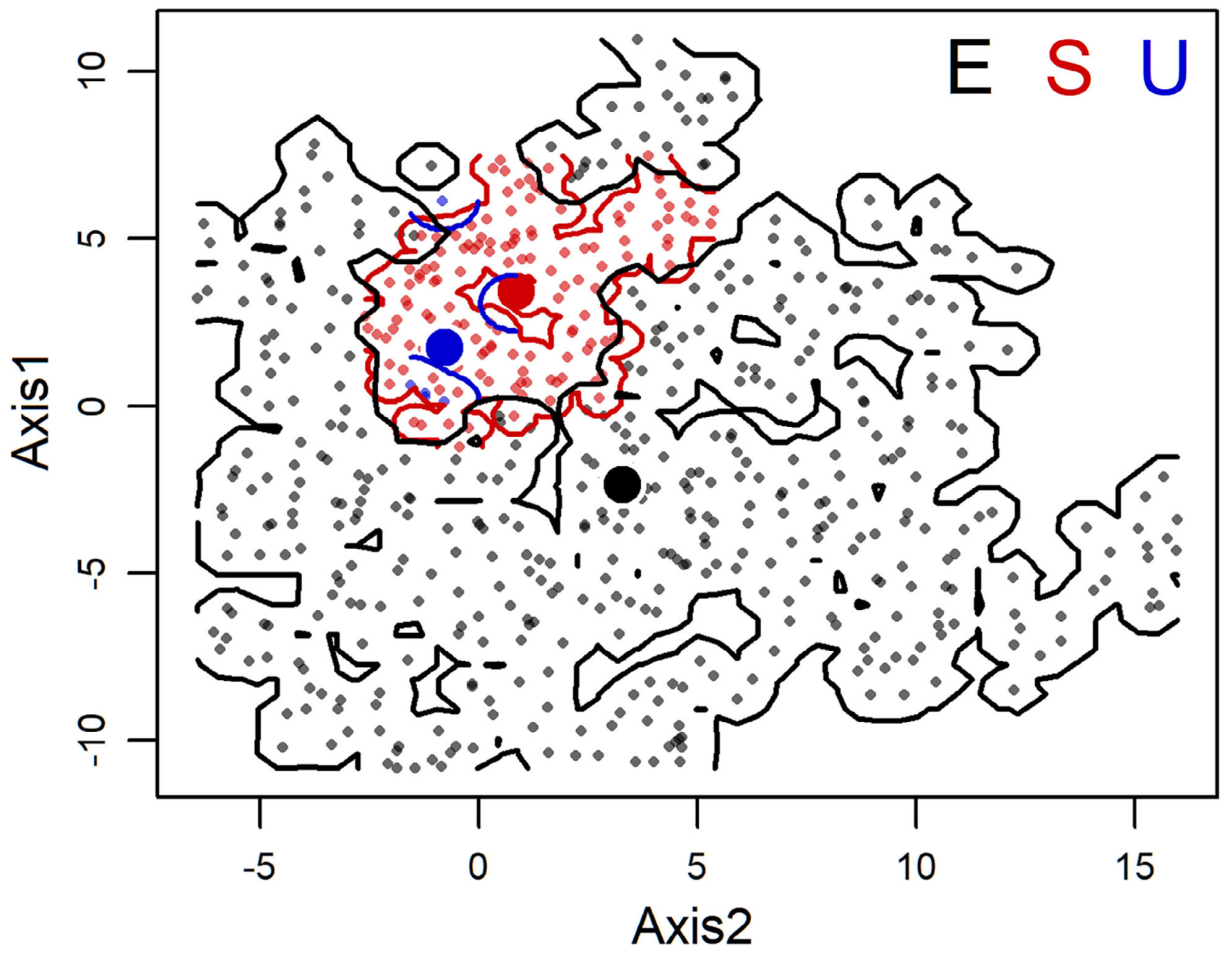
The niche dynamics between native and introduced Tasmanian blue gum. The small red and gray points indicated native and introduced occurrence records, respectively. The niche expansion black lines or blocks), stability (red lines or blocks), and un‐filling (blue lines or blocks) were ca. 263.569, 36.916, and 3.516, respectively. The bigger points represented their centroids.

Our niche estimation showed that niche area of native and introduced Tasmanian blue gum was 40.431 and 300.485, respectively. Niche expansion, stability, and unfilling between native and introduced were 263.569, 36.916, and 3.516, respectively (Figure 4). The *NAR* was 7.432, that is, the niche area of introduced Tasmanian blue gum was approximately more than 7 times that of its native counterpart (Figure 4). The niche similarity index was 0.217, which indicated that the niche positions of introduced and native Tasmanian blue gum were different (Figure 4). Therefore, niche conservatism was rejected.

### 3.3 Spatial patterns of the potential ranges of native and introduced Tasmanian blue gum and their shifts

All seven individual ENMs for native and introduced Tasmanian blue gum had TSS, AUC, and Kappa values that exceeded their respective thresholds (Appendix S4); thus, all seven ENMs were incorporated into the ensemble ENMs for native and introduced Tasmanian blue gum. The TSS, AUC, and Kappa of the integrated ENMs for native (introduced) Tasmanian blue gum were 0.999 (0.905), 0.999 (0.991), and 0.997 (0.870), respectively.

**FIGURE 5:**
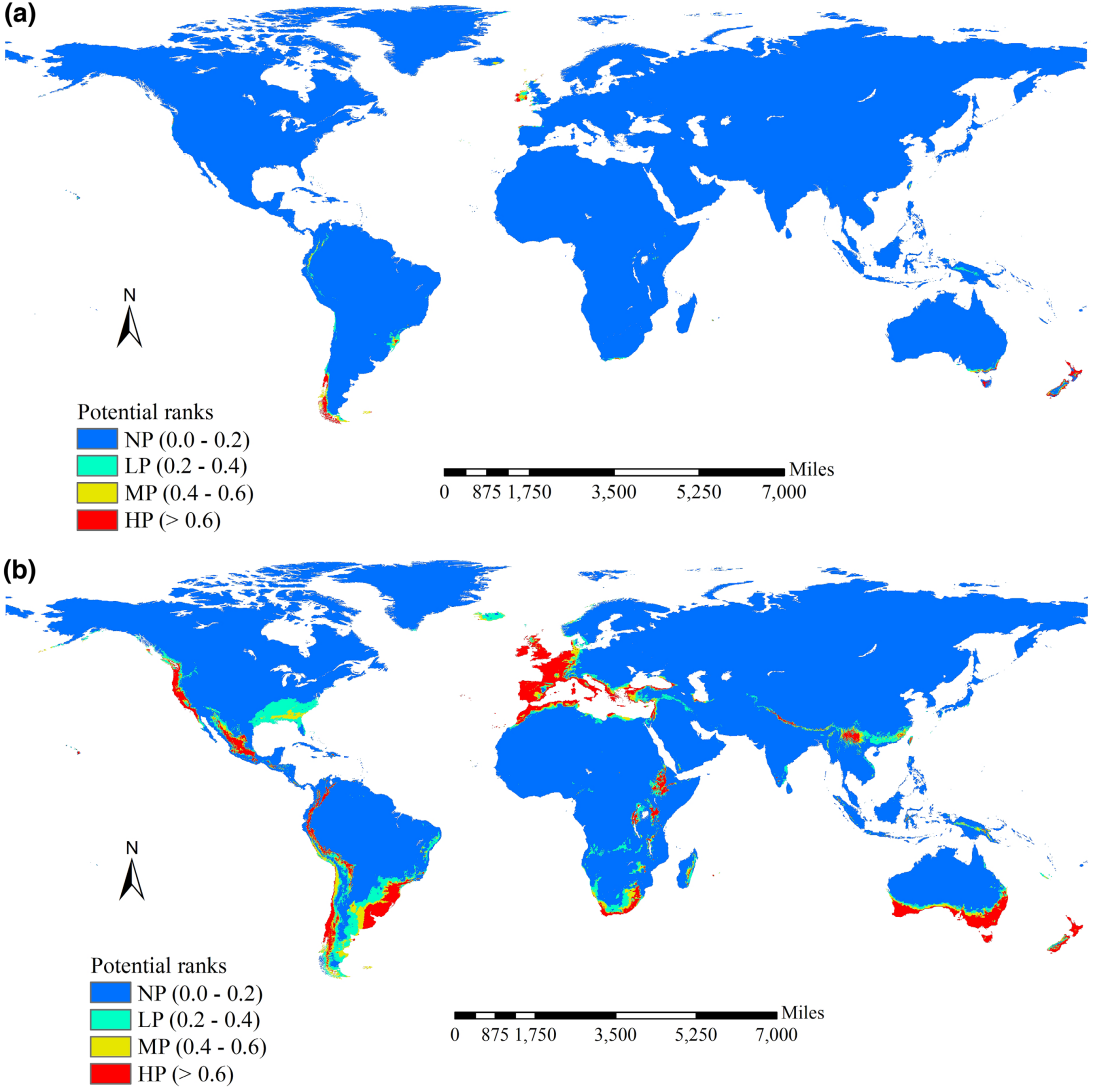
Habitat suitability of Tasmanian blue gum derived from native and introduced occurrence records. High potential habitats derived from native occurrence records were mainly observed in its native regions in Austria, West Europe, west and south coast regions of Chile, as well as southeast regions of Brazil (a). Moderate and low potential habitats showed similar patterns with those of the high ones (a). High potential habitats for introduced Tasmanian blue gum were mainly observed in South Australia, Tasmania islands, New Zealand, South Africa, Ethiopia, Uganda, coast regions of the northern Africa, the low regions at southern slope of the Himalaya, Southwest China, West Europe, west regions of South America and North America, as well as the southeast part of the South America (b). Moderate and low potential habitats showed similar patterns with those of the high ones, except the low potential habitats in south part of the united |states of the America (b). No potential habitats derived both from native and introduced occurrence records were observed in vast regions around the world (a, b).

**FIGURE 6:**
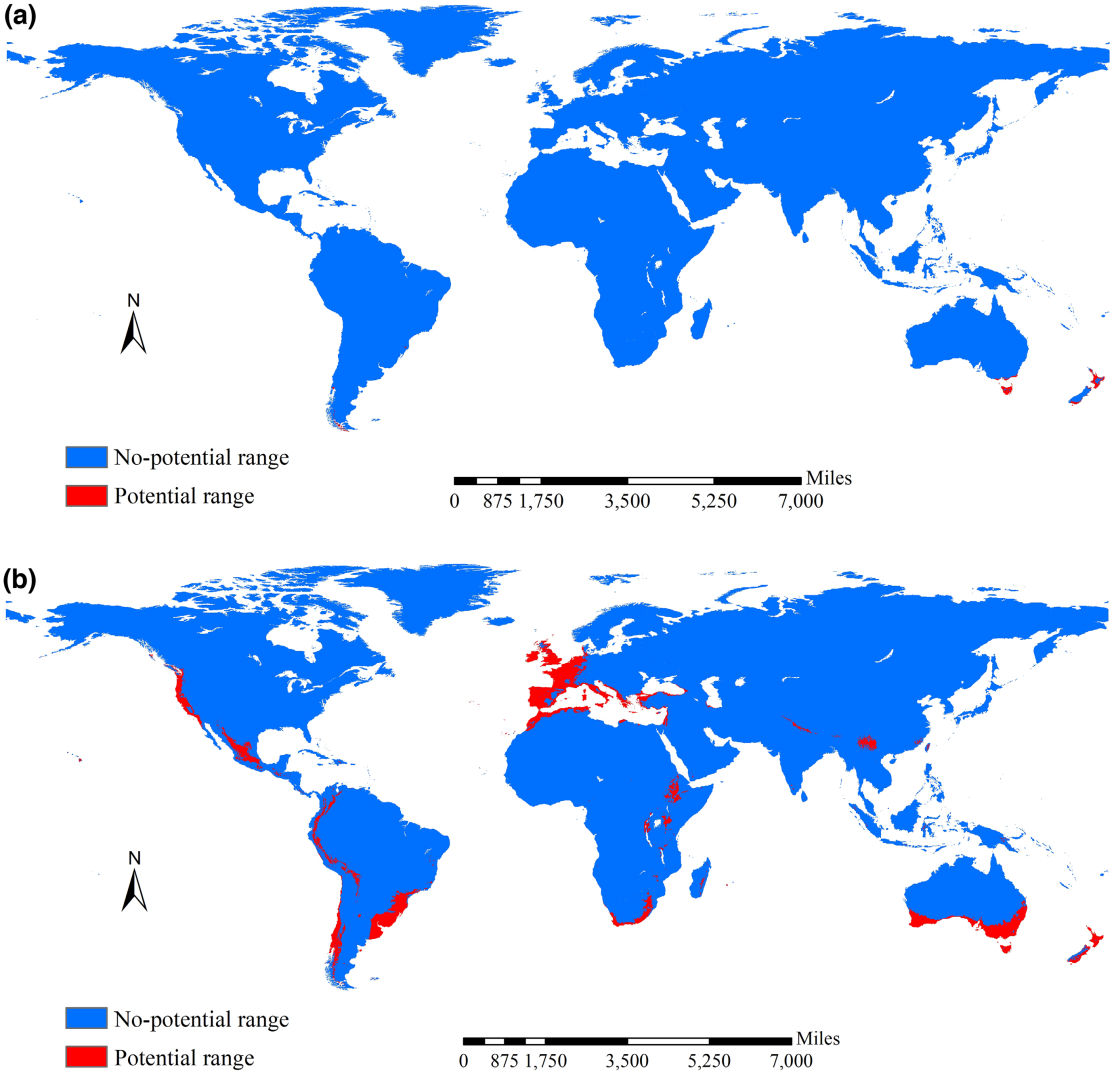
Potential ranges of Tasmanian blue gum derived from native and introduced occurrence records. The potential ranges of native Tasmanian blue gum (the threshold: 0.875) were mainly observed in its native regions (a), and those for introduced Tasmanian blue gum (the threshold: 0.570) were mainly observed in South Australia, Tasmania islands, New Zealand, South Africa, Ethiopia, Uganda, coast regions of the northern Africa, the low regions at southern slope of the Himalaya, Southwest China, West Europe, west regions of South America and North America, as well as the southeast part of the South America (b).

High suitability for native Tasmanian blue gum was mainly observed in its native regions in Australia, western Europe, western and southern coastal regions of Chile, and southeastern regions of Brazil. Patterns of moderate and low suitability were similar to patterns of high suitability. Unsuitable habitats were observed in vast regions (Figure 5). High suitability for introduced Tasmanian blue gum was mainly observed in South Australia, Tasmanian islands, New Zealand, South Africa, Ethiopia, Uganda, coastal regions of northern Africa, the low regions of the southern slope of the Himalayas, Southwest China, western Europe, western and southeastern regions of South America, and North America (Figure 5). Patterns in moderate and low suitability areas were mainly observed in the periphery of high suitability areas; the one exception was the low suitability areas in the southern portion of the United States (Figure 5). Unsuitable areas were observed in vast regions (Figure 5). The potential range of native Tasmanian blue gum (MSS threshold: 0.875) mainly occurred in areas where it is native and the southern tips of South America; the potential range of introduced Tasmanian blue gum (the MSS threshold: 0.570) was mostly in areas of high suitability (Figure 6).

The native potential range of Tasmanian blue gum covered ca. 204,512 km^2^ and spanned 19.60° and 250.88° in latitude and longitude, respectively (Figure 6), whereas the introduced potential range covered ca. 6,409,355 km^2^ and spanned 117.60° and 333.76° in latitude and longitude, respectively (Figure 6). GOF analysis revealed low degrees of spatial concordance between native and introduced potential ranges (0.014), and between native and introduced no‐potential ranges of Tasmanian blue gum (0.244). The spatial changes between native and introduced potential ranges mainly stemmed from northwestward shifts (i.e., expansions of the introduced potential range into Asia, Africa, Europe, South America, and North America), whereas the native potential range was mainly in the southern part of Oceania (Figure 6).

## 4 DISCUSSION

Tasmanian blue gum is a globally cultivated tree, and its niche space might be strongly affected by human activities (e.g., introduction and cultivation). One of the important aspects of the niche shifts between native and introduced Tasmanian blue gum is that the latter mainly tended to occupy niche space in climates with higher max temperatures of the warmest month, mean temperature of the warmest quarter, precipitation of the wettest quarter and precipitation of the wettest month (Figure 3), suggesting that introduced Tasmanian blue gum tends to occupy warmer and wetter climates. This is also consistent with our observation that Tasmanian blue gum is mostly cultivated in exotic regions with warmer and wetter climatic conditions relative to their native regions. This indicates that human‐induced invasions of Tasmanian blue gum have resulted in niche shifts between native and introduced ranges.

The value of niche expansion for Tasmanian blue gum was 262.830, indicating that it has high potential to acclimate to new environments and invade new regions with climatic conditions that differ from those of its native region. We also observed that the introduced niche area was larger than that of its native counterpart. These differences might be related to its introduction and cultivation by humans, as this can reduce dispersal limitation and provide introduced Tasmanian blue gum increased opportunities to acclimate and establish populations in novel climatic conditions that would not have been possible without human intervention. Additionally, cultivation measures, such as weedkilling and the extermination of insect pests, can reduce the abundance of competitors of introduced Tasmanian blue gum, thereby facilitating its expansion in niche space. Moreover, cultivation measures, such as fertilization and irrigation, can aid the survival of introduced Tasmanian blue gum under climatic conditions different from those in their native range. Therefore, Tasmanian blue gum occupied its realized and fundamental niche space, which indicates that its niche space was larger than that of its native counterpart; however, whether this represents a change to the realized niche or whether evolution is involved remains unclear. This also suggests that our conclusions should be interpreted with caution.

Geographic range size reflects species' resource use, and several studies have observed a positive correlation between niche breadth and range size; however, the opposite pattern has also been observed (Bernard et al., 2021; Jourdan et al., 2021; Kafaei et al., 2021; Moore et al., 2018; Slatyer et al., 2013). Therefore, the relationship between niche shifts and range shifts in biological invasions requires more attention. Our results showed that niche area and potential range size increased by ca. 640% and 3100%, respectively, in introduced Tasmanian blue gum relative to its native counterpart. We also observed considerable differences between the model‐predicted global ranges and the actual ranges occupied by the species. This suggests that even small shifts in niche expansion can result in considerable range shifts. Therefore, niche shifts might be a highly sensitive indicator of the potential for range shifts, and invasive species showing greater niche shifts might require increased attention. Generally, range shifts are used to assess the invasion of invasive species (Gong et al., 2020; Liu, Wang, et al., 2020). Our findings indicate that the niche shifts of an invasive species could be used to evaluate invasion risk; the latter might even be more sensitive than range shifts.

Tasmanian blue gum is different from most non‐cultivated invasive plant species. Human introduction and cultivation can result in the breakdown of its dispersal barriers and provide Tasmanian blue gum abundant opportunities to colonize regions beyond its native range, acclimate to novel climatic conditions via the aid of anthropogenic ecological effects (e.g., fertilization and irrigation), and undergo niche shifts. The probability of most invasive plant species being introduced to new areas and cultivated is generally lower than that of Tasmanian blue gum, thus reducing the probability of niche shifts in other species. Accordingly, we observed that introduced and native Tasmanian blue gum occupied different niche positions; niche area of the introduced Tasmanian blue gum was larger than that of the native counterpart, which indicates that niche conservatism is rejected. This suggests that the niche of introduced Tasmanian blue gum has been not conserved relative to that of its native counterpart, which is not consistent with most invasive plant species (Liu, Wolter, et al., 2020). Although controversy remains regarding the generality of niche conservatism, there might be some exceptions for cultivated alien invasive species, probably due to strong modification of niche shifts by human cultivation (Yang et al., 2021).

Niche conservatism is a key factor affecting what inferences can be made from ENMs; if niche conservatism is confirmed, ENMs based on native occurrence records can be used to predict potential distributions under novel and exotic climatic conditions as well as future climate change scenarios. Although niche conservatism was confirmed in most alien invasive species (e.g., Liu, Wolter, et al., 2020; Petitpierre et al., 2012), we found that introduced Tasmanian blue gum has been not conserved relative to that of its native counterpart. It may imply that though for most alien invasive species, the ENMs projection for their potential range may be reliable or applicable, caution is needed when we use ENMs to predict potential range of cultivated invasive species.

Finally, Tasmanian blue gum is native to a relatively small island with relatively low variation in climate compared with variation in climate on a global scale. Thus, interpretation of the results of our study is complex because the niche area of the native climatic niche is small. Tasmanian blue gum might thrive in warmer conditions in its native range, but such conditions do not occur in Tasmania. This can have a substantial effect on our ability to extrapolate their ranges and elucidate the mechanisms underlying range expansion. Careful interpretation of our findings is essential to maximize the utility of Tasmanian blue gum as a case study.

## 5 CONCLUSIONS

The ecological niche concept has been used to provide insight into various topics in ecology and biogeography. Whether cultivated invasive species, whose niches are strongly modified by human activities, conserve the climatic niches of their native counterparts remains unclear. Our results rejected niche conservatism in the climatic niches of introduced Tasmanian blue gum; moreover, the niche area of introduced Tasmanian blue gum was ca. 7.4 times that of its native counterpart, as it can survive in hotter, colder, wetter, and drier climates. Given that small increases in niche area can result in large changes in range size, native and introduced occurrence records should be used to predict potential ranges, although our interpretation of the data should be taken with caution.

## AUTHOR CONTRIBUTIONS

**Runyao Cao:** Data curation (equal); formal analysis (lead); investigation (lead); software (lead); writing – original draft (equal). **Xiang Gong:** Data curation (equal); formal analysis (supporting); investigation (supporting); software (supporting). **Jianmeng Feng:** Conceptualization (lead); funding acquisition (lead); methodology (lead); project administration (lead); supervision (lead); writing – original draft (equal). **Rujing Yang:** Data curation (lead); formal analysis (supporting); investigation (supporting); software (supporting).

## CONFLICT OF INTEREST

The authors declare that they have no known competing financial interests or personal relationships that could have appeared to influence the work reported in this paper.

## Supporting information


Appendix S1

Appendix S2

Appendix S3

Appendix S4

Appendix S5

## Data Availability

R code for niche and range shifts, and global dataset of occurrence records were available at https://doi.org/10.5061/dryad.37pvmcvpf.
