# Supplementary material for: Niche and range dynamics of Tasmanian blue gum (Eucalyptus globulus Labill.), a globally cultivated invasive tree
Source: Ecol Evol. 2022 Sep 17;12(9):e9305. doi: 10.1002/ece3.9305 (PMC9482005; doi:10.1002/ece3.9305)
Supplement: Supplementary file 1 — Appendix S1 [file ECE3-12-e9305-s001.docx]

| S1 Correlations among the 19 predictors   \|  \| S1-1 Correlations among the 19 predictors in native Tasmanian blue gum **(N=398)** \| \| \| \| \| \| \| \| \| \| \| \| \| \| \| \| \| \| \| \| \| \| \| --- \| --- \| --- \| --- \| --- \| --- \| --- \| --- \| --- \| --- \| --- \| --- \| --- \| --- \| --- \| --- \| --- \| --- \| --- \| --- \| --- \| --- \| --- \| \|  \| \|  \| bio9 \| bio8 \| bio7 \| bio6 \| bio5 \| bio4 \| bio13 \| bio14 \| bio15 \| bio16 \| bio17 \| bio12 \| bio3 \| bio11 \| bio2 \| bio10 \| bio19 \| bio1 \| bio18 \| Note \| \| bio9 \| \| Pearson Correlation \| 1.00 \| -0.255** \| -0.228** \| 0.648** \| 0.361** \| -0.10 \| 0.272** \| -0.106* \| 0.445** \| 0.290** \| 0.117* \| 0.270** \| -0.568** \| 0.584** \| -0.369** \| 0.536** \| 0.349** \| 0.553** \| -0.09 \|  \| \| Sig. (2-tailed) \|  \| 0.00 \| 0.00 \| 0.00 \| 0.00 \| 0.05 \| 0.00 \| 0.03 \| 0.00 \| 0.00 \| 0.02 \| 0.00 \| 0.00 \| 0.00 \| 0.00 \| 0.00 \| 0.00 \| 0.00 \| 0.08 \|  \| \| bio8 \| \| Pearson Correlation \| -0.255** \| 1.00 \| 0.385** \| 0.00 \| 0.447** \| 0.242** \| -0.642** \| -0.725** \| -0.265** \| -0.647** \| -0.713** \| -0.704** \| 0.515** \| 0.339** \| 0.474** \| 0.437** \| -0.678** \| 0.425** \| -0.598** \|  \| \| Sig. (2-tailed) \| 0.00 \|  \| 0.00 \| 0.94 \| 0.00 \| 0.00 \| 0.00 \| 0.00 \| 0.00 \| 0.00 \| 0.00 \| 0.00 \| 0.00 \| 0.00 \| 0.00 \| 0.00 \| 0.00 \| 0.00 \| 0.00 \|  \| \| bio7 \| \| Pearson Correlation \| -0.228** \| 0.385** \| 1.00 \| -0.565** \| 0.608** \| 0.942** \| -0.460** \| -0.331** \| -0.438** \| -0.443** \| -0.305** \| -0.408** \| 0.657** \| -0.208** \| 0.966** \| 0.223** \| -0.493** \| 0.03 \| -0.171** \|  \| \| Sig. (2-tailed) \| 0.00 \| 0.00 \|  \| 0.00 \| 0.00 \| 0.00 \| 0.00 \| 0.00 \| 0.00 \| 0.00 \| 0.00 \| 0.00 \| 0.00 \| 0.00 \| 0.00 \| 0.00 \| 0.00 \| 0.50 \| 0.00 \|  \| \| bio6 \| \| Pearson Correlation \| 0.648** \| 0.00 \| -0.565** \| 1.00 \| 0.312** \| -0.475** \| 0.257** \| -0.213** \| 0.663** \| 0.264** \| -0.112* \| 0.166** \| -0.687** \| 0.894** \| -0.646** \| 0.664** \| 0.317** \| 0.779** \| -0.266** \|  \| \| Sig. (2-tailed) \| 0.00 \| 0.94 \| 0.00 \|  \| 0.00 \| 0.00 \| 0.00 \| 0.00 \| 0.00 \| 0.00 \| 0.03 \| 0.00 \| 0.00 \| 0.00 \| 0.00 \| 0.00 \| 0.00 \| 0.00 \| 0.00 \|  \| \| bio5 \| \| Pearson Correlation \| 0.361** \| 0.447** \| 0.608** \| 0.312** \| 1.00 \| 0.627** \| -0.283** \| -0.586** \| 0.134** \| -0.256** \| -0.459** \| -0.311** \| 0.10 \| 0.620** \| 0.490** \| 0.896** \| -0.263** \| 0.788** \| -0.453** \|  \| \| Sig. (2-tailed) \| 0.00 \| 0.00 \| 0.00 \| 0.00 \|  \| 0.00 \| 0.00 \| 0.00 \| 0.01 \| 0.00 \| 0.00 \| 0.00 \| 0.06 \| 0.00 \| 0.00 \| 0.00 \| 0.00 \| 0.00 \| 0.00 \|  \| \| bio4 \| \| Pearson Correlation \| -0.10 \| 0.242** \| 0.942** \| -0.475** \| 0.627** \| 1.00 \| -0.390** \| -0.319** \| -0.345** \| -0.361** \| -0.270** \| -0.326** \| 0.399** \| -0.196** \| 0.834** \| 0.265** \| -0.394** \| 0.05 \| -0.163** \|  \| \| Sig. (2-tailed) \| 0.05 \| 0.00 \| 0.00 \| 0.00 \| 0.00 \|  \| 0.00 \| 0.00 \| 0.00 \| 0.00 \| 0.00 \| 0.00 \| 0.00 \| 0.00 \| 0.00 \| 0.00 \| 0.00 \| 0.30 \| 0.00 \|  \| \| bio13 \| \| Pearson Correlation \| 0.272** \| -0.642** \| -0.460** \| 0.257** \| -0.283** \| -0.390** \| 1.00 \| 0.744** \| 0.704** \| 0.997** \| 0.851** \| 0.983** \| -0.546** \| -0.04 \| -0.531** \| -0.212** \| 0.988** \| -0.159** \| 0.791** \|  \| \| Sig. (2-tailed) \| 0.00 \| 0.00 \| 0.00 \| 0.00 \| 0.00 \| 0.00 \|  \| 0.00 \| 0.00 \| 0.00 \| 0.00 \| 0.00 \| 0.00 \| 0.44 \| 0.00 \| 0.00 \| 0.00 \| 0.00 \| 0.00 \|  \| \| bio14 \| \| Pearson Correlation \| -0.106* \| -0.725** \| -0.331** \| -0.213** \| -0.586** \| -0.319** \| 0.744** \| 1.00 \| 0.112* \| 0.732** \| 0.911** \| 0.807** \| -0.193** \| -0.507** \| -0.324** \| -0.640** \| 0.712** \| -0.602** \| 0.919** \|  \| \| Sig. (2-tailed) \| 0.03 \| 0.00 \| 0.00 \| 0.00 \| 0.00 \| 0.00 \| 0.00 \|  \| 0.03 \| 0.00 \| 0.00 \| 0.00 \| 0.00 \| 0.00 \| 0.00 \| 0.00 \| 0.00 \| 0.00 \| 0.00 \|  \| \| bio15 \| \| Pearson Correlation \| 0.445** \| -0.265** \| -0.438** \| 0.663** \| 0.134** \| -0.345** \| 0.704** \| 0.112* \| 1.00 \| 0.714** \| 0.246** \| 0.595** \| -0.669** \| 0.474** \| -0.542** \| 0.310** \| 0.718** \| 0.370** \| 0.165** \|  \| \| Sig. (2-tailed) \| 0.00 \| 0.00 \| 0.00 \| 0.00 \| 0.01 \| 0.00 \| 0.00 \| 0.03 \|  \| 0.00 \| 0.00 \| 0.00 \| 0.00 \| 0.00 \| 0.00 \| 0.00 \| 0.00 \| 0.00 \| 0.00 \|  \| \| bio16 \| \| Pearson Correlation \| 0.290** \| -0.647** \| -0.443** \| 0.264** \| -0.256** \| -0.361** \| 0.997** \| 0.732** \| 0.714** \| 1.00 \| 0.840** \| 0.985** \| -0.567** \| -0.03 \| -0.524** \| -0.193** \| 0.992** \| -0.147** \| 0.781** \|  \| \| Sig. (2-tailed) \| 0.00 \| 0.00 \| 0.00 \| 0.00 \| 0.00 \| 0.00 \| 0.00 \| 0.00 \| 0.00 \|  \| 0.00 \| 0.00 \| 0.00 \| 0.51 \| 0.00 \| 0.00 \| 0.00 \| 0.00 \| 0.00 \|  \| \| bio17 \| \| Pearson Correlation \| 0.117* \| -0.713** \| -0.305** \| -0.112* \| -0.459** \| -0.270** \| 0.851** \| 0.911** \| 0.246** \| 0.840** \| 1.00 \| 0.915** \| -0.276** \| -0.369** \| -0.334** \| -0.480** \| 0.828** \| -0.456** \| 0.955** \|  \| \| Sig. (2-tailed) \| 0.02 \| 0.00 \| 0.00 \| 0.03 \| 0.00 \| 0.00 \| 0.00 \| 0.00 \| 0.00 \| 0.00 \|  \| 0.00 \| 0.00 \| 0.00 \| 0.00 \| 0.00 \| 0.00 \| 0.00 \| 0.00 \|  \| \| bio12 \| \| Pearson Correlation \| 0.270** \| -0.704** \| -0.408** \| 0.166** \| -0.311** \| -0.326** \| 0.983** \| 0.807** \| 0.595** \| 0.985** \| 0.915** \| 1.00 \| -0.519** \| -0.133** \| -0.486** \| -0.275** \| 0.980** \| -0.239** \| 0.854** \|  \| \| Sig. (2-tailed) \| 0.00 \| 0.00 \| 0.00 \| 0.00 \| 0.00 \| 0.00 \| 0.00 \| 0.00 \| 0.00 \| 0.00 \| 0.00 \|  \| 0.00 \| 0.01 \| 0.00 \| 0.00 \| 0.00 \| 0.00 \| 0.00 \|  \| \| bio3 \| \| Pearson Correlation \| -0.568** \| 0.515** \| 0.657** \| -0.687** \| 0.10 \| 0.399** \| -0.546** \| -0.193** \| -0.669** \| -0.567** \| -0.276** \| -0.519** \| 1.00 \| -0.347** \| 0.828** \| -0.167** \| -0.626** \| -0.224** \| -0.115* \|  \| \| Sig. (2-tailed) \| 0.00 \| 0.00 \| 0.00 \| 0.00 \| 0.06 \| 0.00 \| 0.00 \| 0.00 \| 0.00 \| 0.00 \| 0.00 \| 0.00 \|  \| 0.00 \| 0.00 \| 0.00 \| 0.00 \| 0.00 \| 0.02 \|  \| \| bio11 \| \| Pearson Correlation \| 0.584** \| 0.339** \| -0.208** \| 0.894** \| 0.620** \| -0.196** \| -0.04 \| -0.507** \| 0.474** \| -0.03 \| -0.369** \| -0.133** \| -0.347** \| 1.00 \| -0.263** \| 0.893** \| 0.00 \| 0.968** \| -0.481** \|  \| \| Sig. (2-tailed) \| 0.00 \| 0.00 \| 0.00 \| 0.00 \| 0.00 \| 0.00 \| 0.44 \| 0.00 \| 0.00 \| 0.51 \| 0.00 \| 0.01 \| 0.00 \|  \| 0.00 \| 0.00 \| 0.96 \| 0.00 \| 0.00 \|  \| \| bio2 \| \| Pearson Correlation \| -0.369** \| 0.474** \| 0.966** \| -0.646** \| 0.490** \| 0.834** \| -0.531** \| -0.324** \| -0.542** \| -0.524** \| -0.334** \| -0.486** \| 0.828** \| -0.263** \| 1.00 \| 0.118* \| -0.583** \| -0.04 \| -0.176** \|  \| \| Sig. (2-tailed) \| 0.00 \| 0.00 \| 0.00 \| 0.00 \| 0.00 \| 0.00 \| 0.00 \| 0.00 \| 0.00 \| 0.00 \| 0.00 \| 0.00 \| 0.00 \| 0.00 \|  \| 0.02 \| 0.00 \| 0.41 \| 0.00 \|  \| \| bio10 \| \| Pearson Correlation \| 0.536** \| 0.437** \| 0.223** \| 0.664** \| 0.896** \| 0.265** \| -0.212** \| -0.640** \| 0.310** \| -0.193** \| -0.480** \| -0.275** \| -0.167** \| 0.893** \| 0.118* \| 1.00 \| -0.172** \| 0.975** \| -0.544** \|  \| \| Sig. (2-tailed) \| 0.00 \| 0.00 \| 0.00 \| 0.00 \| 0.00 \| 0.00 \| 0.00 \| 0.00 \| 0.00 \| 0.00 \| 0.00 \| 0.00 \| 0.00 \| 0.00 \| 0.02 \|  \| 0.00 \| 0.00 \| 0.00 \|  \| \| bio19 \| \| Pearson Correlation \| 0.349** \| -0.678** \| -0.493** \| 0.317** \| -0.263** \| -0.394** \| 0.988** \| 0.712** \| 0.718** \| 0.992** \| 0.828** \| 0.980** \| -0.626** \| 0.00 \| -0.583** \| -0.172** \| 1.00 \| -0.121* \| 0.749** \|  \| \| Sig. (2-tailed) \| 0.00 \| 0.00 \| 0.00 \| 0.00 \| 0.00 \| 0.00 \| 0.00 \| 0.00 \| 0.00 \| 0.00 \| 0.00 \| 0.00 \| 0.00 \| 0.96 \| 0.00 \| 0.00 \|  \| 0.02 \| 0.00 \|  \| \| bio1 \| \| Pearson Correlation \| 0.553** \| 0.425** \| 0.03 \| 0.779** \| 0.788** \| 0.05 \| -0.159** \| -0.602** \| 0.370** \| -0.147** \| -0.456** \| -0.239** \| -0.224** \| 0.968** \| -0.04 \| 0.975** \| -0.121* \| 1.00 \| -0.539** \|  \| \| Sig. (2-tailed) \| 0.00 \| 0.00 \| 0.50 \| 0.00 \| 0.00 \| 0.30 \| 0.00 \| 0.00 \| 0.00 \| 0.00 \| 0.00 \| 0.00 \| 0.00 \| 0.00 \| 0.41 \| 0.00 \| 0.02 \|  \| 0.00 \|  \| \| bio18 \| \| Pearson Correlation \| -0.09 \| -0.598** \| -0.171** \| -0.266** \| -0.453** \| -0.163** \| 0.791** \| 0.919** \| 0.165** \| 0.781** \| 0.955** \| 0.854** \| -0.115* \| -0.481** \| -0.176** \| -0.544** \| 0.749** \| -0.539** \| 1.00 \|  \| \| Sig. (2-tailed) \| 0.08 \| 0.00 \| 0.00 \| 0.00 \| 0.00 \| 0.00 \| 0.00 \| 0.00 \| 0.00 \| 0.00 \| 0.00 \| 0.00 \| 0.02 \| 0.00 \| 0.00 \| 0.00 \| 0.00 \| 0.00 \|  \|  \|   S1-2 Correlations among the 19 predictors in introduced Tasmanian blue gum (N=2090) | | | | | | | | | | | | | | | | | | | | | |  | |  | |
| --- | --- | --- | --- | --- | --- | --- | --- | --- | --- | --- | --- | --- | --- | --- | --- | --- | --- | --- | --- | --- | --- | --- | --- | --- | --- | --- | --- | --- | --- | --- | --- | --- | --- | --- | --- | --- | --- | --- | --- | --- | --- | --- | --- | --- | --- | --- | --- | --- | --- | --- | --- | --- | --- | --- | --- | --- | --- | --- | --- | --- | --- | --- | --- | --- | --- | --- | --- | --- | --- | --- | --- | --- | --- | --- | --- | --- | --- | --- | --- | --- | --- | --- | --- | --- | --- | --- | --- | --- | --- | --- | --- | --- | --- | --- | --- | --- | --- | --- | --- | --- | --- | --- | --- | --- | --- | --- | --- | --- | --- | --- | --- | --- | --- | --- | --- | --- | --- | --- | --- | --- | --- | --- | --- | --- | --- | --- | --- | --- | --- | --- | --- | --- | --- | --- | --- | --- | --- | --- | --- | --- | --- | --- | --- | --- | --- | --- | --- | --- | --- | --- | --- | --- | --- | --- | --- | --- | --- | --- | --- | --- | --- | --- | --- | --- | --- | --- | --- | --- | --- | --- | --- | --- | --- | --- | --- | --- | --- | --- | --- | --- | --- | --- | --- | --- | --- | --- | --- | --- | --- | --- | --- | --- | --- | --- | --- | --- | --- | --- | --- | --- | --- | --- | --- | --- | --- | --- | --- | --- | --- | --- | --- | --- | --- | --- | --- | --- | --- | --- | --- | --- | --- | --- | --- | --- | --- | --- | --- | --- | --- | --- | --- | --- | --- | --- | --- | --- | --- | --- | --- | --- | --- | --- | --- | --- | --- | --- | --- | --- | --- | --- | --- | --- | --- | --- | --- | --- | --- | --- | --- | --- | --- | --- | --- | --- | --- | --- | --- | --- | --- | --- | --- | --- | --- | --- | --- | --- | --- | --- | --- | --- | --- | --- | --- | --- | --- | --- | --- | --- | --- | --- | --- | --- | --- | --- | --- | --- | --- | --- | --- | --- | --- | --- | --- | --- | --- | --- | --- | --- | --- | --- | --- | --- | --- | --- | --- | --- | --- | --- | --- | --- | --- | --- | --- | --- | --- | --- | --- | --- | --- | --- | --- | --- | --- | --- | --- | --- | --- | --- | --- | --- | --- | --- | --- | --- | --- | --- | --- | --- | --- | --- | --- | --- | --- | --- | --- | --- | --- | --- | --- | --- | --- | --- | --- | --- | --- | --- | --- | --- | --- | --- | --- | --- | --- | --- | --- | --- | --- | --- | --- | --- | --- | --- | --- | --- | --- | --- | --- | --- | --- | --- | --- | --- | --- | --- | --- | --- | --- | --- | --- | --- | --- | --- | --- | --- | --- | --- | --- | --- | --- | --- | --- | --- | --- | --- | --- | --- | --- | --- | --- | --- | --- | --- | --- | --- | --- | --- | --- | --- | --- | --- | --- | --- | --- | --- | --- | --- | --- | --- | --- | --- | --- | --- | --- | --- | --- | --- | --- | --- | --- | --- | --- | --- | --- | --- | --- | --- | --- | --- | --- | --- | --- | --- | --- | --- | --- | --- | --- | --- | --- | --- | --- | --- | --- | --- | --- | --- | --- | --- | --- | --- | --- | --- | --- | --- | --- | --- | --- | --- | --- | --- | --- | --- | --- | --- | --- | --- | --- | --- | --- | --- | --- | --- | --- | --- | --- | --- | --- | --- | --- | --- | --- | --- | --- | --- | --- | --- | --- | --- | --- | --- | --- | --- | --- | --- | --- | --- | --- | --- | --- | --- | --- | --- | --- | --- | --- | --- | --- | --- | --- | --- | --- | --- | --- | --- | --- | --- | --- | --- | --- | --- | --- | --- | --- | --- | --- | --- | --- | --- | --- | --- | --- | --- | --- | --- | --- | --- | --- | --- | --- | --- | --- | --- | --- | --- | --- | --- | --- | --- | --- | --- | --- | --- | --- | --- | --- | --- | --- | --- | --- | --- | --- | --- | --- | --- | --- | --- | --- | --- | --- | --- | --- | --- | --- | --- | --- | --- | --- | --- | --- | --- | --- | --- | --- | --- | --- | --- | --- | --- | --- | --- | --- | --- | --- | --- | --- | --- | --- | --- | --- | --- | --- | --- | --- | --- | --- | --- | --- | --- | --- | --- | --- | --- | --- | --- | --- | --- | --- | --- | --- | --- | --- | --- | --- | --- | --- | --- | --- | --- | --- | --- | --- | --- | --- | --- | --- | --- | --- | --- | --- | --- | --- | --- | --- | --- | --- | --- | --- | --- | --- | --- | --- | --- | --- | --- | --- | --- | --- | --- | --- | --- | --- | --- | --- | --- | --- | --- | --- | --- | --- | --- | --- | --- | --- | --- | --- | --- | --- | --- | --- | --- | --- | --- | --- | --- | --- | --- | --- | --- | --- | --- | --- | --- | --- | --- | --- | --- | --- | --- | --- | --- | --- | --- | --- | --- | --- | --- | --- | --- | --- | --- | --- | --- | --- | --- | --- | --- | --- | --- | --- | --- | --- | --- | --- | --- | --- | --- | --- | --- | --- | --- | --- | --- | --- | --- | --- | --- | --- | --- | --- | --- | --- | --- | --- | --- | --- | --- | --- | --- | --- | --- | --- | --- | --- | --- | --- | --- | --- | --- | --- | --- | --- | --- | --- | --- | --- | --- | --- | --- | --- | --- | --- | --- | --- | --- | --- | --- | --- | --- | --- | --- | --- | --- | --- | --- | --- | --- | --- | --- | --- | --- | --- | --- | --- | --- | --- | --- | --- | --- | --- | --- | --- | --- | --- | --- | --- | --- | --- | --- | --- | --- | --- | --- | --- | --- | --- | --- | --- | --- | --- | --- | --- | --- | --- | --- | --- | --- | --- | --- | --- | --- | --- | --- | --- | --- | --- | --- | --- | --- | --- | --- | --- | --- | --- | --- | --- | --- | --- | --- | --- | --- | --- | --- | --- | --- | --- | --- | --- | --- | --- | --- | --- | --- | --- | --- | --- | --- | --- | --- | --- | --- | --- | --- | --- | --- | --- | --- | --- |
|  |  | bio9 | bio8 | bio7 | bio16 | bio17 | bio15 | bio6 | bio14 | bio5 | bio13 | bio4 | bio12 | bio3 | bio11 | bio2 | bio10 | bio19 | bio1 | bio18 | Note | |  | |  |
| bio9 | Pearson Correlation | 1.00 | -.065^**^ | .208^**^ | -.050^*^ | -.430^**^ | .345^**^ | .465^**^ | -.456^**^ | .612^**^ | -.050^*^ | .211^**^ | -.239^**^ | -.138^**^ | .413^**^ | .070^**^ | .612^**^ | .146^**^ | .546^**^ | -.508^**^ |  | |  | |  |
|  | Sig. (2-tailed) |  | 0.00 | 0.00 | 0.02 | 0.00 | 0.00 | 0.00 | 0.00 | 0.00 | 0.02 | 0.00 | 0.00 | 0.00 | 0.00 | 0.00 | 0.00 | 0.00 | 0.00 | 0.00 |  | |  | |  |
| bio8 | Pearson Correlation | -.065^**^ | 1.00 | 0.00 | .113^**^ | -.132^**^ | .210^**^ | .485^**^ | -.123^**^ | .394^**^ | .154^**^ | -.112^**^ | #### | .201^**^ | .660^**^ | .142^**^ | .551^**^ | -.436^**^ | .726^**^ | .436^**^ |  | |  | |  |
|  | Sig. (2-tailed) | 0.00 |  | 0.95 | 0.00 | 0.00 | 0.00 | 0.00 | 0.00 | 0.00 | 0.00 | 0.00 | 0.26 | 0.00 | 0.00 | 0.00 | 0.00 | 0.00 | 0.00 | 0.00 |  | |  | |  |
| bio7 | Pearson Correlation | .208^**^ | 0.00 | 1.00 | -.295^**^ | -.261^**^ | .095^**^ | -.512^**^ | -.240^**^ | .720^**^ | -.278^**^ | .825^**^ | -.400^**^ | -.357^**^ | -.305^**^ | .631^**^ | .502^**^ | -.296^**^ | .106^**^ | -.240^**^ |  | |  | |  |
|  | Sig. (2-tailed) | 0.00 | 0.95 |  | 0.00 | 0.00 | 0.00 | 0.00 | 0.00 | 0.00 | 0.00 | 0.00 | 0.00 | 0.00 | 0.00 | 0.00 | 0.00 | 0.00 | 0.00 | 0.00 |  | |  | |  |
| bio16 | Pearson Correlation | -.050^*^ | .113^**^ | -.295^**^ | 1.00 | .141^**^ | .338^**^ | .257^**^ | .099^**^ | -.127^**^ | .991^**^ | -.306^**^ | .835^**^ | .219^**^ | .252^**^ | -.106^**^ | -.050^*^ | .637^**^ | .134^**^ | .461^**^ |  | |  | |  |
|  | Sig. (2-tailed) | 0.02 | 0.00 | 0.00 |  | 0.00 | 0.00 | 0.00 | 0.00 | 0.00 | 0.00 | 0.00 | 0.00 | 0.00 | 0.00 | 0.00 | 0.02 | 0.00 | 0.00 | 0.00 |  | |  | |  |
| bio17 | Pearson Correlation | -.430^**^ | -.132^**^ | -.261^**^ | .141^**^ | 1.00 | -.794^**^ | -.230^**^ | .986^**^ | -.481^**^ | .102^**^ | -.053^*^ | .629^**^ | -.182^**^ | -.377^**^ | -.411^**^ | -.419^**^ | .207^**^ | -.442^**^ | .588^**^ |  | |  | |  |
|  | Sig. (2-tailed) | 0.00 | 0.00 | 0.00 | 0.00 |  | 0.00 | 0.00 | 0.00 | 0.00 | 0.00 | 0.02 | 0.00 | 0.00 | 0.00 | 0.00 | 0.00 | 0.00 | 0.00 | 0.00 |  | |  | |  |
| bio15 | Pearson Correlation | .345^**^ | .210^**^ | .095^**^ | .338^**^ | -.794^**^ | 1.00 | .326^**^ | -.788^**^ | .371^**^ | .376^**^ | -.146^**^ | -.186^**^ | .356^**^ | .507^**^ | .402^**^ | .354^**^ | .091^**^ | .497^**^ | -.282^**^ |  | |  | |  |
|  | Sig. (2-tailed) | 0.00 | 0.00 | 0.00 | 0.00 | 0.00 |  | 0.00 | 0.00 | 0.00 | 0.00 | 0.00 | 0.00 | 0.00 | 0.00 | 0.00 | 0.00 | 0.00 | 0.00 | 0.00 |  | |  | |  |
| bio6 | Pearson Correlation | .465^**^ | .485^**^ | -.512^**^ | .257^**^ | -.230^**^ | .326^**^ | 1.00 | -.242^**^ | .228^**^ | .265^**^ | -.472^**^ | .097^**^ | .277^**^ | .903^**^ | -.262^**^ | .437^**^ | .110^**^ | .760^**^ | 0.02 |  | |  | |  |
|  | Sig. (2-tailed) | 0.00 | 0.00 | 0.00 | 0.00 | 0.00 | 0.00 |  | 0.00 | 0.00 | 0.00 | 0.00 | 0.00 | 0.00 | 0.00 | 0.00 | 0.00 | 0.00 | 0.00 | 0.32 |  | |  | |  |
| bio14 | Pearson Correlation | -.456^**^ | -.123^**^ | -.240^**^ | .099^**^ | .986^**^ | -.788^**^ | -.242^**^ | 1.00 | -.467^**^ | .060^**^ | -.049^*^ | .578^**^ | -.174^**^ | -.381^**^ | -.372^**^ | -.419^**^ | .166^**^ | -.441^**^ | .571^**^ |  | |  | |  |
|  | Sig. (2-tailed) | 0.00 | 0.00 | 0.00 | 0.00 | 0.00 | 0.00 | 0.00 |  | 0.00 | 0.01 | 0.03 | 0.00 | 0.00 | 0.00 | 0.00 | 0.00 | 0.00 | 0.00 | 0.00 |  | |  | |  |
| bio5 | Pearson Correlation | .612^**^ | .394^**^ | .720^**^ | -.127^**^ | -.481^**^ | .371^**^ | .228^**^ | -.467^**^ | 1.00 | -.101^**^ | .553^**^ | -.375^**^ | -.181^**^ | .384^**^ | .503^**^ | .922^**^ | -.247^**^ | .735^**^ | -.254^**^ |  | |  | |  |
|  | Sig. (2-tailed) | 0.00 | 0.00 | 0.00 | 0.00 | 0.00 | 0.00 | 0.00 | 0.00 |  | 0.00 | 0.00 | 0.00 | 0.00 | 0.00 | 0.00 | 0.00 | 0.00 | 0.00 | 0.00 |  | |  | |  |
| bio13 | Pearson Correlation | -.050^*^ | .154^**^ | -.278^**^ | .991^**^ | .102^**^ | .376^**^ | .265^**^ | .060^**^ | -.101^**^ | 1.00 | -.306^**^ | .805^**^ | .247^**^ | .277^**^ | -.076^**^ | -0.03 | .597^**^ | .165^**^ | .463^**^ |  | |  | |  |
|  | Sig. (2-tailed) | 0.02 | 0.00 | 0.00 | 0.00 | 0.00 | 0.00 | 0.00 | 0.01 | 0.00 |  | 0.00 | 0.00 | 0.00 | 0.00 | 0.00 | 0.25 | 0.00 | 0.00 | 0.00 |  | |  | |  |
| bio4 | Pearson Correlation | .211^**^ | -.112^**^ | .825^**^ | -.306^**^ | -.053^*^ | -.146^**^ | -.472^**^ | -.049^*^ | .553^**^ | -.306^**^ | 1.00 | -.263^**^ | -.784^**^ | -.484^**^ | .098^**^ | .493^**^ | -.135^**^ | -0.02 | -.231^**^ |  | |  | |  |
|  | Sig. (2-tailed) | 0.00 | 0.00 | 0.00 | 0.00 | 0.02 | 0.00 | 0.00 | 0.03 | 0.00 | 0.00 |  | 0.00 | 0.00 | 0.00 | 0.00 | 0.00 | 0.00 | 0.39 | 0.00 |  | |  | |  |
| bio12 | Pearson Correlation | -.239^**^ | -0.02 | -.400^**^ | .835^**^ | .629^**^ | -.186^**^ | .097^**^ | .578^**^ | -.375^**^ | .805^**^ | -.263^**^ | 1.00 | .047^*^ | -0.01 | -.361^**^ | -.268^**^ | .648^**^ | -.146^**^ | .636^**^ |  | |  | |  |
|  | Sig. (2-tailed) | 0.00 | 0.26 | 0.00 | 0.00 | 0.00 | 0.00 | 0.00 | 0.00 | 0.00 | 0.00 | 0.00 |  | 0.03 | 0.55 | 0.00 | 0.00 | 0.00 | 0.00 | 0.00 |  | |  | |  |
| bio3 | Pearson Correlation | -.138^**^ | .201^**^ | -.357^**^ | .219^**^ | -.182^**^ | .356^**^ | .277^**^ | -.174^**^ | -.181^**^ | .247^**^ | -.784^**^ | .047^*^ | 1.00 | .492^**^ | .466^**^ | -.277^**^ | -0.02 | .156^**^ | .156^**^ |  | |  | |  |
|  | Sig. (2-tailed) | 0.00 | 0.00 | 0.00 | 0.00 | 0.00 | 0.00 | 0.00 | 0.00 | 0.00 | 0.00 | 0.00 | 0.03 |  | 0.00 | 0.00 | 0.00 | 0.34 | 0.00 | 0.00 |  | |  | |  |
| bio11 | Pearson Correlation | .413^**^ | .660^**^ | -.305^**^ | .252^**^ | -.377^**^ | .507^**^ | .903^**^ | -.381^**^ | .384^**^ | .277^**^ | -.484^**^ | #### | .492^**^ | 1.00 | .113^**^ | .522^**^ | -.085^**^ | .880^**^ | .086^**^ |  | |  | |  |
|  | Sig. (2-tailed) | 0.00 | 0.00 | 0.00 | 0.00 | 0.00 | 0.00 | 0.00 | 0.00 | 0.00 | 0.00 | 0.00 | 0.55 | 0.00 |  | 0.00 | 0.00 | 0.00 | 0.00 | 0.00 |  | |  | |  |
| bio2 | Pearson Correlation | .070^**^ | .142^**^ | .631^**^ | -.106^**^ | -.411^**^ | .402^**^ | -.262^**^ | -.372^**^ | .503^**^ | -.076^**^ | .098^**^ | -.361^**^ | .466^**^ | .113^**^ | 1.00 | .210^**^ | -.290^**^ | .208^**^ | -.140^**^ |  | |  | |  |
|  | Sig. (2-tailed) | 0.00 | 0.00 | 0.00 | 0.00 | 0.00 | 0.00 | 0.00 | 0.00 | 0.00 | 0.00 | 0.00 | 0.00 | 0.00 | 0.00 |  | 0.00 | 0.00 | 0.00 | 0.00 |  | |  | |  |
| bio10 | Pearson Correlation | .612^**^ | .551^**^ | .502^**^ | -.050^*^ | -.419^**^ | .354^**^ | .437^**^ | -.419^**^ | .922^**^ | -0.03 | .493^**^ | -.268^**^ | -.277^**^ | .522^**^ | .210^**^ | 1.00 | -.218^**^ | .858^**^ | -.137^**^ |  | |  | |  |
|  | Sig. (2-tailed) | 0.00 | 0.00 | 0.00 | 0.02 | 0.00 | 0.00 | 0.00 | 0.00 | 0.00 | 0.25 | 0.00 | 0.00 | 0.00 | 0.00 | 0.00 |  | 0.00 | 0.00 | 0.00 |  | |  | |  |
| bio19 | Pearson Correlation | .146^**^ | -.436^**^ | -.296^**^ | .637^**^ | .207^**^ | .091^**^ | .110^**^ | .166^**^ | -.247^**^ | .597^**^ | -.135^**^ | .648^**^ | -0.02 | -.085^**^ | -.290^**^ | -.218^**^ | 1.00 | -.192^**^ | -.112^**^ |  | |  | |  |
|  | Sig. (2-tailed) | 0.00 | 0.00 | 0.00 | 0.00 | 0.00 | 0.00 | 0.00 | 0.00 | 0.00 | 0.00 | 0.00 | 0.00 | 0.34 | 0.00 | 0.00 | 0.00 |  | 0.00 | 0.00 |  | |  | |  |
| bio1 | Pearson Correlation | .546^**^ | .726^**^ | .106^**^ | .134^**^ | -.442^**^ | .497^**^ | .760^**^ | -.441^**^ | .735^**^ | .165^**^ | -0.02 | -.146^**^ | .156^**^ | .880^**^ | .208^**^ | .858^**^ | -.192^**^ | 1.00 | 0.02 |  | |  | |  |
|  | Sig. (2-tailed) | 0.00 | 0.00 | 0.00 | 0.00 | 0.00 | 0.00 | 0.00 | 0.00 | 0.00 | 0.00 | 0.39 | 0.00 | 0.00 | 0.00 | 0.00 | 0.00 | 0.00 |  | 0.46 |  | |  | |  |
| bio18 | Pearson Correlation | -.508^**^ | .436^**^ | -.240^**^ | .461^**^ | .588^**^ | -.282^**^ | 0.02 | .571^**^ | -.254^**^ | .463^**^ | -.231^**^ | .636^**^ | .156^**^ | .086^**^ | -.140^**^ | -.137^**^ | -.112^**^ | 0.02 | 1.00 |  | |  | |  |
|  | Sig. (2-tailed) | 0.00 | 0.00 | 0.00 | 0.00 | 0.00 | 0.00 | 0.32 | 0.00 | 0.00 | 0.00 | 0.00 | 0.00 | 0.00 | 0.00 | 0.00 | 0.00 | 0.00 | 0.46 | 0.00 |  | |  | |  |
|  |  |  |  |  |  |  |  |  |  |  |  |  |  |  |  |  |  |  |  |  |  | |  | |  |
|  |  |  |  |  |  |  |  |  |  |  |  |  |  |  |  |  |  |  |  |  |  | |  | |  |
|  |  |  |  |  |  |  |  |  |  |  |  |  |  |  |  |  |  |  |  |  |  | |  | |  |
|  |  |  |  |  |  |  |  |  |  |  |  |  |  |  |  |  |  |  |  |  |  | |  | |  |
|  |  |  |  |  |  |  |  |  |  |  |  |  |  |  |  |  |  |  |  |  |  | |  | |  |
|  |  |  |  |  |  |  |  |  |  |  |  |  |  |  |  |  |  |  |  |  |  | |  | |  |
|  |  |  |  |  |  |  |  |  |  |  |  |  |  |  |  |  |  |  |  |  |  | |  | |  |
|  |  |  |  |  |  |  |  |  |  |  |  |  |  |  |  |  |  |  |  |  |  | |  | |  |
|  |  |  |  |  |  |  |  |  |  |  |  |  |  |  |  |  |  |  |  |  |  | |  | |  |
|  |  |  |  |  |  |  |  |  |  |  |  |  |  |  |  |  |  |  |  |  |  | |  | |  |
|  |  |  |  |  |  |  |  |  |  |  |  |  |  |  |  |  |  |  |  |  |  | |  | |  |
|  |  |  |  |  |  |  |  |  |  |  |  |  |  |  |  |  |  |  |  |  |  | |  | |  |
