# Supplementary material for: Niche and range dynamics of Tasmanian blue gum (Eucalyptus globulus Labill.), a globally cultivated invasive tree
Source: Ecol Evol. 2022 Sep 17;12(9):e9305. doi: 10.1002/ece3.9305 (PMC9482005; doi:10.1002/ece3.9305)
Supplement: Supplementary file 2 — Appendix S2 [file ECE3-12-e9305-s003.docx]

| Native Tasmanian blue gum | | Introduced Tasmanian blue gum | |
| --- | --- | --- | --- |
| Predictors | Importance values | Predictors | Importance values |
| bio4 | 0.33 | bio1 | 0.27 |
| bio17 | 0.22 | bio11 | 0.26 |
| bio3 | 0.11 | bio4 | 0.22 |
| bio15 | 0.09 | bio6 | 0.10 |
| bio11 | 0.09 | bio12 | 0.09 |
| bio19 | 0.09 | bio19 | 0.07 |
| bio18 | 0.06 | bio10 | 0.07 |
| bio12 | 0.06 | bio7 | 0.06 |
| bio1 | 0.05 | bio18 | 0.05 |
| bio10 | 0.05 | bio17 | 0.05 |
| bio6 | 0.05 | bio16 | 0.05 |
| bio16 | 0.05 | bio3 | 0.04 |
| bio13 | 0.04 | bio5 | 0.04 |
| bio7 | 0.03 | bio2 | 0.03 |
| bio14 | 0.03 | bio13 | 0.03 |
| bio5 | 0.03 | bio9 | 0.03 |
| bio8 | 0.01 | bio14 | 0.02 |
| bio9 | 0.01 | bio8 | 0.02 |
| bio2 | 0.01 | bio15 | 0.01 |

S2 Importance values of climatic predictors in the preliminary ecological niche models
