# Supplementary material for: Niche and range dynamics of Tasmanian blue gum (Eucalyptus globulus Labill.), a globally cultivated invasive tree
Source: Ecol Evol. 2022 Sep 17;12(9):e9305. doi: 10.1002/ece3.9305 (PMC9482005; doi:10.1002/ece3.9305)
Supplement: Supplementary file 3 — Appendix S3 [file ECE3-12-e9305-s005.docx]

S3 Climatic predictors in the final ecological niche models and their importance values

| Native Tasmanian blue gum | | Introduced Tasmanian blue gum | | |
| --- | --- | --- | --- | --- |
| Predictors | Importance values | Predictors | Importance values |  |
| Temperature Seasonality (Bio4) | 0.43 | Annual Mean Temperature (Bio1) | 0.37 |  |
| Precipitation Of Driest Quarter (Bio17) | 0.20 | Temperature Seasonality (Bio4) | 0.35 |  |
| Mean Temperature Of Coldest Quarter (Bio11) | 0.15 | Precipitation Of Coldest Quarter (Bio19) | 0.10 |  |
| Precipitation Seasonality (Bio15) | 0.15 | Mean Temperature Of Driest Quarter (Bio9) | 0.07 |  |
| Isothermality (Bio3) | 0.14 | Annual Precipitation Bio12 | 0.07 |  |
| Max Temperature Of Warmest Month (Bio5) | 0.11 | Precipitation Of Warmest Quarter (Bio18) | 0.05 |  |
| Mean Temperature Of Driest Quarter (Bio9) | 0.06 | Precipitation Of Driest Quarter Bio (17) | 0.03 |  |
|  |  | Mean Diurnal Range (Bio2) | 0.02 |  |
