# Supplementary material for: Niche and range dynamics of Tasmanian blue gum (Eucalyptus globulus Labill.), a globally cultivated invasive tree
Source: Ecol Evol. 2022 Sep 17;12(9):e9305. doi: 10.1002/ece3.9305 (PMC9482005; doi:10.1002/ece3.9305)
Supplement: Supplementary file 4 — Appendix S4 [file ECE3-12-e9305-s002.pdf]

S4 Evaluation metrics in the final ecological niche models

S4-1 Evaluation metrics in the final ecological niche models for native Tasmanian blue gum

ANN Testing.data.RUN.PA

	RUN1.PA1	.RUN2.PA1	RUN3.PA1	RUN4.PA1	RUN5.PA1	RUN1.PA2	RUN2.PA2	RUN3.PA2	RUN4.PA2	RUN5.PA2	RUN1.PA3	RUN2.PA3	RUN3.PA3	RUN4.PA3	RUN5.PA3	RUN1.PA4	RUN2.PA4	RUN3.PA4	RUN4.PA4	RUN5.PA4	RUN1.PA5	RUN2.PA5	RUN3.PA5	RUN4.PA5	RUN5.PA5
TSS	0.987	0.997	1	0.997	0.993	1	0.993	0.993	1	1	0.993	0.993	0.993	1	0.997	0.993	0.993	0.997	0.987	0.985	0.997	0.973	1	0.987	1
ROC	0.996	1	1	1	0.998	1	1	0.998	1	1	1	1	0.998	1	1	0.997	0.997	0.999	0.995	0.996	1	0.999	1	1	1
KAPPA	0.982	0.994	1	0.994	0.988	1	0.988	0.988	1	1	0.988	0.988	0.988	1	0.994	0.988	0.988	0.994	0.977	0.982	0.994	0.959	1	0.977	1

CTA

	RUN1.PA1	.RUN2.PA1	RUN3.PA1	RUN4.PA1	RUN5.PA1	RUN1.PA2	RUN2.PA2	RUN3.PA2	RUN4.PA2	RUN5.PA2	RUN1.PA3	RUN2.PA3	RUN3.PA3	RUN4.PA3	RUN5.PA3	RUN1.PA4	RUN2.PA4	RUN3.PA4	RUN4.PA4	RUN5.PA4	RUN1.PA5	RUN2.PA5	RUN3.PA5	RUN4.PA5	RUN5.PA5
TSS	0.982	0.997	0.958	0.98	0.98	0.982	0.987	0.99	0.971	0.987	0.99	0.982	0.988	0.993	0.99	0.965	0.983	0.987	0.978	0.997	1	0.977	0.988	0.997	0.992
ROC	0.991	0.998	0.981	0.99	0.99	0.991	0.993	0.995	0.987	0.993	0.995	0.991	0.994	0.997	0.995	0.982	0.992	0.993	0.989	0.998	1	0.988	0.994	0.998	0.996
KAPPA	0.977	0.994	0.953	0.965	0.965	0.977	0.977	0.983	0.976	0.977	0.983	0.977	0.988	0.988	0.983	0.965	0.988	0.977	0.971	0.994	1	0.977	0.988	0.994	0.994

FDA

	RUN1.PA1	.RUN2.PA1	RUN3.PA1	RUN4.PA1	RUN5.PA1	RUN1.PA2	RUN2.PA2	RUN3.PA2	RUN4.PA2	RUN5.PA2	RUN1.PA3	RUN2.PA3	RUN3.PA3	RUN4.PA3	RUN5.PA3	RUN1.PA4	RUN2.PA4	RUN3.PA4	RUN4.PA4	RUN5.PA4	RUN1.PA5	RUN2.PA5	RUN3.PA5	RUN4.PA5	RUN5.PA5
TSS	0.99	1	0.983	0.99	0.987	0.997	0.997	0.987	1	1	0.993	0.993	0.993	0.997	0.987	0.99	0.997	0.997	0.99	0.993	1	1	0.99	0.993	1
ROC	0.995	1	0.993	0.995	0.993	0.998	0.998	0.997	1	1	0.998	0.997	1	0.998	0.993	0.995	0.998	0.998	0.995	0.997	1	1	0.998	0.997	1
KAPPA	0.983	1	0.971	0.983	0.977	0.994	0.994	0.977	1	1	0.988	0.988	0.988	0.994	0.977	0.983	0.994	0.994	0.983	0.988	1	1	0.983	0.988	1

GBM

	RUN1.PA1	.RUN2.PA1	RUN3.PA1	RUN4.PA1	RUN5.PA1	RUN1.PA2	RUN2.PA2	RUN3.PA2	RUN4.PA2	RUN5.PA2	RUN1.PA3	RUN2.PA3	RUN3.PA3	RUN4.PA3	RUN5.PA3	RUN1.PA4	RUN2.PA4	RUN3.PA4	RUN4.PA4	RUN5.PA4	RUN1.PA5	RUN2.PA5	RUN3.PA5	RUN4.PA5	RUN5.PA5
TSS	0.99	1	0.961	0.993	0.99	0.987	0.993	0.99	0.997	0.993	0.997	0.99	0.997	0.997	0.997	0.968	1	0.997	0.99	0.997	1	0.993	0.993	0.997	0.993
ROC	0.995	1	0.997	1	1	0.998	1	1	1	1	1	1	1	1	1	0.998	1	1	0.998	1	1	1	0.998	0.998	1
KAPPA	0.983	1	0.959	0.988	0.983	0.977	0.988	0.983	0.994	0.988	0.994	0.983	0.994	0.994	0.994	0.971	1	0.994	0.983	0.994	1	0.988	0.988	0.994	0.994

GLM.

	RUN1.PA1	.RUN2.PA1	RUN3.PA1	RUN4.PA1	RUN5.PA1	RUN1.PA2	RUN2.PA2	RUN3.PA2	RUN4.PA2	RUN5.PA2	RUN1.PA3	RUN2.PA3	RUN3.PA3	RUN4.PA3	RUN5.PA3	RUN1.PA4	RUN2.PA4	RUN3.PA4	RUN4.PA4	RUN5.PA4	RUN1.PA5	RUN2.PA5	RUN3.PA5	RUN4.PA5	RUN5.PA5
TSS	0.993	1	0.993	0.993	0.997	0.997	0.99	0.993	0.943	0.983	1	0.997	1	1	0.983	0.99	0.997	0.987	0.993	0.953	0.997	0.997	0.99	0.987	0.993
ROC	0.997	1	0.997	0.997	0.998	0.998	0.995	0.997	0.972	0.992	1	0.998	1	1	0.993	0.995	0.998	0.993	0.997	0.977	0.998	0.998	0.995	0.993	0.997
KAPPA	0.988	1	0.988	0.988	0.994	0.994	0.983	0.988	0.92	0.971	1	0.994	1	1	0.971	0.983	0.994	0.977	0.988	0.953	0.994	0.994	0.983	0.977	0.988

.MAXENT.Phillips

	RUN1.PA1	.RUN2.PA1	RUN3.PA1	RUN4.PA1	RUN5.PA1	RUN1.PA2	RUN2.PA2	RUN3.PA2	RUN4.PA2	RUN5.PA2	RUN1.PA3	RUN2.PA3	RUN3.PA3	RUN4.PA3	RUN5.PA3	RUN1.PA4	RUN2.PA4	RUN3.PA4	RUN4.PA4	RUN5.PA4	RUN1.PA5	RUN2.PA5	RUN3.PA5	RUN4.PA5	RUN5.PA5
TSS	0.953	0.982	0.983	0.955	0.958	0.967	0.978	0.977	0.978	0.945	0.963	0.98	0.985	0.993	0.987	0.963	0.98	0.983	0.97	0.96	1	0.977	0.963	0.987	0.993
ROC	0.978	0.996	0.992	0.977	0.979	0.982	0.989	0.988	0.988	0.969	0.982	0.99	0.997	0.997	0.993	0.982	0.998	0.992	0.985	0.98	1	0.988	0.982	0.993	0.997
KAPPA	0.937	0.977	0.971	0.931	0.937	0.959	0.971	0.96	0.971	0.947	0.937	0.965	0.982	0.988	0.977	0.937	0.982	0.971	0.948	0.932	1	0.96	0.937	0.977	0.988

RF

	RUN1.PA1	.RUN2.PA1	RUN3.PA1	RUN4.PA1	RUN5.PA1	RUN1.PA2	RUN2.PA2	RUN3.PA2	RUN4.PA2	RUN5.PA2	RUN1.PA3	RUN2.PA3	RUN3.PA3	RUN4.PA3	RUN5.PA3	RUN1.PA4	RUN2.PA4	RUN3.PA4	RUN4.PA4	RUN5.PA4	RUN1.PA5	RUN2.PA5	RUN3.PA5	RUN4.PA5	RUN5.PA5
TSS	0.99	1	0.99	1	0.993	1	1	1	1	1	1	0.99	1	1	1	0.997	1	1	0.997	1	1	0.997	0.997	1	1
ROC	1	1	1	1	1	1	1	1	1	1	1	1	1	1	1	1	1	1	1	1	1	1	1	1	1
KAPPA	0.988	1	0.983	1	0.988	1	1	1	1	1	1	0.988	1	1	1	0.994	1	1	0.994	1	1	0.994	0.994	1	1

S4-2 Evaluation metrics in the final ecological niche models for introduced Tasmanian blue gum

ANN Testing.data.RUN.PA

	RUN1.PA1	.RUN2.PA1	RUN3.PA1	RUN4.PA1	RUN5.PA1	RUN1.PA2	RUN2.PA2	RUN3.PA2	RUN4.PA2	RUN5.PA2	RUN1.PA3	RUN2.PA3	RUN3.PA3	RUN4.PA3	RUN5.PA3	RUN1.PA4	RUN2.PA4	RUN3.PA4	RUN4.PA4	RUN5.PA4	RUN1.PA5	RUN2.PA5	RUN3.PA5	RUN4.PA5	RUN5.PA5
TSS	0.895	0.895	0.831	0.82	0.753	0.841	0.825	0.833	0.801	0.837	0.844	0.833	0.821	0.856	0.856	0.861	0.813	0.85	0.794	0.799	0.82	0.802	0.856	0.82	0.848
ROC	0.98	0.981	0.958	0.922	0.916	0.956	0.948	0.931	0.946	0.954	0.965	0.955	0.949	0.943	0.955	0.976	0.951	0.951	0.92	0.939	0.961	0.954	0.956	0.949	0.95
KAPPA	0.895	0.895	0.831	0.82	0.753	0.841	0.825	0.833	0.801	0.837	0.844	0.833	0.821	0.856	0.856	0.861	0.813	0.85	0.794	0.799	0.82	0.802	0.856	0.82	0.848

CTA

	RUN1.PA1	.RUN2.PA1	RUN3.PA1	RUN4.PA1	RUN5.PA1	RUN1.PA2	RUN2.PA2	RUN3.PA2	RUN4.PA2	RUN5.PA2	RUN1.PA3	RUN2.PA3	RUN3.PA3	RUN4.PA3	RUN5.PA3	RUN1.PA4	RUN2.PA4	RUN3.PA4	RUN4.PA4	RUN5.PA4	RUN1.PA5	RUN2.PA5	RUN3.PA5	RUN4.PA5	RUN5.PA5
TSS	0.893	0.888	0.885	0.884	0.869	0.879	0.876	0.863	0.893	0.887	0.866	0.856	0.86	0.898	0.868	0.858	0.884	0.892	0.877	0.877	0.888	0.869	0.882	0.871	0.874
ROC	0.966	0.969	0.965	0.961	0.966	0.959	0.957	0.96	0.967	0.969	0.954	0.966	0.965	0.971	0.963	0.953	0.968	0.968	0.96	0.965	0.979	0.956	0.967	0.952	0.961
KAPPA	0.893	0.888	0.885	0.884	0.869	0.879	0.876	0.863	0.893	0.887	0.866	0.856	0.86	0.898	0.868	0.858	0.884	0.892	0.877	0.877	0.888	0.869	0.882	0.871	0.874

FDA

	RUN1.PA1	.RUN2.PA1	RUN3.PA1	RUN4.PA1	RUN5.PA1	RUN1.PA2	RUN2.PA2	RUN3.PA2	RUN4.PA2	RUN5.PA2	RUN1.PA3	RUN2.PA3	RUN3.PA3	RUN4.PA3	RUN5.PA3	RUN1.PA4	RUN2.PA4	RUN3.PA4	RUN4.PA4	RUN5.PA4	RUN1.PA5	RUN2.PA5	RUN3.PA5	RUN4.PA5	RUN5.PA5
TSS	0.879	0.888	0.89	0.879	0.876	0.896	0.868	0.876	0.887	0.893	0.866	0.871	0.868	0.89	0.88	0.86	0.866	0.871	0.871	0.864	0.887	0.868	0.877	0.861	0.88
ROC	0.973	0.979	0.981	0.97	0.969	0.979	0.97	0.975	0.976	0.979	0.974	0.975	0.978	0.984	0.976	0.972	0.976	0.98	0.977	0.977	0.98	0.977	0.976	0.968	0.977
KAPPA	0.879	0.888	0.89	0.879	0.876	0.896	0.868	0.876	0.887	0.893	0.866	0.871	0.868	0.89	0.88	0.86	0.866	0.871	0.871	0.864	0.887	0.868	0.877	0.861	0.88

GBM

	RUN1.PA1	.RUN2.PA1	RUN3.PA1	RUN4.PA1	RUN5.PA1	RUN1.PA2	RUN2.PA2	RUN3.PA2	RUN4.PA2	RUN5.PA2	RUN1.PA3	RUN2.PA3	RUN3.PA3	RUN4.PA3	RUN5.PA3	RUN1.PA4	RUN2.PA4	RUN3.PA4	RUN4.PA4	RUN5.PA4	RUN1.PA5	RUN2.PA5	RUN3.PA5	RUN4.PA5	RUN5.PA5
TSS	0.896	0.895	0.885	0.888	0.882	0.912	0.885	0.892	0.901	0.904	0.885	0.882	0.887	0.907	0.9	0.882	0.885	0.9	0.885	0.879	0.901	0.872	0.9	0.88	0.89
ROC	0.986	0.988	0.986	0.982	0.982	0.985	0.981	0.984	0.983	0.988	0.98	0.983	0.984	0.989	0.985	0.985	0.985	0.989	0.986	0.986	0.987	0.984	0.982	0.981	0.984
KAPPA	0.896	0.895	0.885	0.888	0.882	0.912	0.885	0.892	0.901	0.904	0.885	0.882	0.887	0.907	0.9	0.882	0.885	0.9	0.885	0.879	0.901	0.872	0.9	0.88	0.89

GLM.

	RUN1.PA1	.RUN2.PA1	RUN3.PA1	RUN4.PA1	RUN5.PA1	RUN1.PA2	RUN2.PA2	RUN3.PA2	RUN4.PA2	RUN5.PA2	RUN1.PA3	RUN2.PA3	RUN3.PA3	RUN4.PA3	RUN5.PA3	RUN1.PA4	RUN2.PA4	RUN3.PA4	RUN4.PA4	RUN5.PA4	RUN1.PA5	RUN2.PA5	RUN3.PA5	RUN4.PA5	RUN5.PA5
TSS	0.893	0.9	0.893	0.882	0.876	0.887	0.868	0.872	0.893	0.895	0.869	0.885	0.893	0.895	0.895	0.871	0.876	0.876	0.869	0.874	0.896	0.876	0.877	0.871	0.898
ROC	0.98	0.985	0.984	0.978	0.977	0.98	0.972	0.981	0.98	0.984	0.976	0.977	0.979	0.984	0.979	0.977	0.98	0.983	0.981	0.98	0.985	0.978	0.98	0.974	0.979
KAPPA	0.893	0.9	0.893	0.882	0.876	0.887	0.868	0.872	0.893	0.895	0.869	0.885	0.893	0.895	0.895	0.871	0.876	0.876	0.869	0.874	0.896	0.876	0.877	0.871	0.898

.MAXENT.Phillips

	RUN1.PA1	.RUN2.PA1	RUN3.PA1	RUN4.PA1	RUN5.PA1	RUN1.PA2	RUN2.PA2	RUN3.PA2	RUN4.PA2	RUN5.PA2	RUN1.PA3	RUN2.PA3	RUN3.PA3	RUN4.PA3	RUN5.PA3	RUN1.PA4	RUN2.PA4	RUN3.PA4	RUN4.PA4	RUN5.PA4	RUN1.PA5	RUN2.PA5	RUN3.PA5	RUN4.PA5	RUN5.PA5
TSS	0.81	0.853	0.828	0.781	0.788	0.848	0.839	0.812	0.839	0.836	0.815	0.812	0.809	0.86	0.815	0.799	0.804	0.794	0.772	0.785	0.852	0.772	0.775	0.78	0.785
ROC	0.91	0.935	0.925	0.897	0.896	0.931	0.922	0.913	0.924	0.926	0.916	0.914	0.909	0.941	0.918	0.902	0.908	0.9	0.891	0.899	0.965	0.894	0.901	0.903	0.907
KAPPA	0.81	0.853	0.828	0.781	0.788	0.848	0.839	0.812	0.839	0.836	0.815	0.812	0.809	0.86	0.815	0.799	0.804	0.794	0.772	0.785	0.852	0.772	0.775	0.78	0.785

RF

	RUN1.PA1	.RUN2.PA1	RUN3.PA1	RUN4.PA1	RUN5.PA1	RUN1.PA2	RUN2.PA2	RUN3.PA2	RUN4.PA2	RUN5.PA2	RUN1.PA3	RUN2.PA3	RUN3.PA3	RUN4.PA3	RUN5.PA3	RUN1.PA4	RUN2.PA4	RUN3.PA4	RUN4.PA4	RUN5.PA4	RUN1.PA5	RUN2.PA5	RUN3.PA5	RUN4.PA5	RUN5.PA5
TSS	0.912	0.919	0.914	0.903	0.903	0.912	0.896	0.895	0.912	0.915	0.888	0.89	0.903	0.92	0.914	0.895	0.909	0.903	0.906	0.909	0.914	0.892	0.917	0.888	0.901
ROC	0.991	0.991	0.991	0.986	0.986	0.99	0.985	0.989	0.989	0.992	0.986	0.986	0.989	0.991	0.988	0.986	0.991	0.991	0.99	0.991	0.988	0.986	0.987	0.984	0.986
KAPPA	0.912	0.919	0.914	0.903	0.903	0.912	0.896	0.895	0.912	0.915	0.888	0.89	0.903	0.92	0.914	0.895	0.909	0.903	0.906	0.909	0.914	0.892	0.917	0.888	0.901
