# Supplementary material for: Niche and range dynamics of Tasmanian blue gum (Eucalyptus globulus Labill.), a globally cultivated invasive tree
Source: Ecol Evol. 2022 Sep 17;12(9):e9305. doi: 10.1002/ece3.9305 (PMC9482005; doi:10.1002/ece3.9305)
Supplement: Supplementary file 5 — Appendix S5 [file ECE3-12-e9305-s004.doc]

S5 The PCA loadings of bioclimatic predictors
Bioclimatic predictors	PCA1	PCA2	
Bio1 (Annual Mean Temperature)	-0.83546570	0.41673222	
Bio2 (Mean Diurnal Range)	-0.54770127	-0.13938041	
Bio3 (Isothermality)	-0.03895951	0.48739688	
Bio4 (Temperature Seasonality)	-0.33715918	-0.61274254	
Bio5 (Max Temperature of Warmest Month)	-0.88729457	-0.08333154	
Bio6 (min temperature of coldest month)	-0.42070238	0.68949947	
Bio7 (Temperature Annual Range)	-0.55513123	-0.53128032	
Bio8 (Mean Temperature of Wettest Quarter)	-0.45347676	0.35016370	
Bio9 (Mean Temperature of Driest Quarter)	-0.66373264	0.06459198	
Bio10 (Mean Temperature of Warmest Quarter)	-0.86394074	0.05244050	
Bio11 (Mean Temperature of Coldest Quarter)	-0.61814542	0.67401762	
Bio12 (Annual Precipitation)	0.55987035	0.64472340	
Bio13 (Precipitation of Wettest Month)	0.08983254	0.81033960	
Bio14 (Precipitation of Driest Month)	0.82279292	0.02323143	
Bio15 (Precipitation Seasonality)	-0.69380673	0.37973828	
Bio16 (Precipitation of Wettest Quarter)	0.13223538	0.80517517	
Bio17 (Precipitation of Driest Quarter)	0.83397736	0.05385638	
Bio18 (Precipitation of Warmest Quarter)	0.44495566	0.43038460	
Bio19 (Precipitation of Coldest Quarter)	0.29787664	0.45905345	
